# Pediatric epilepsy surgery: Global survey of referral and presurgical evaluation practices

**DOI:** 10.1002/epi.70312

**Published:** 2026-06-02

**Authors:** Georgia Ramantani, J. Helen Cross, Dorottya Cserpan, Emtnan Ahemad, Javier Aparicio, Alexis Arzimanoglou, Carmen Barba, William Bingaman, Kees Braun, Richard J. Burman, James Butler, Lixin Cai, Dezhi Cao, Roberto Caraballo, Mathilde Chipaux‐Raffo, Thomas Cloppenborg, Arthur Cukiert, Cristine Mella Cukiert, Sameer Dal, Dario Englot, Noelle Enright, Aria Fallah, Sarah Ferrand Sorbets, Martha Feucht, Deepak Gill, Laura Guio Mahecha, Aris Hadjinicolaou, Tove Hallböök, A. Simon Harvey, Hans Holthausen, Yuwu Jiang, Lakshminarayanan Kannan, Kalman A. Katlowitz, Katarzyna Kotulska‐Jóźwiak, Martin Kudr, Matt Lallas, Ahsan Moosa Naduvil Valappil, Sandeep Bhagwan Patil, Tom Pieper, Nicola Specchio, Manjari Tripathi, Lejla Vendegh, Howard Weiner, Jo Wilmshurst, Mary Lou Smith, Elaine Wirrell, ILAE Pediatric Epilepsy Surgery Task Force and Study Group, Fabrice Bartolomei, Juan Carlos Benedetti Isaac, Poodipedi Sarat Chandra, Mary Connoly, Daniel Curry, Vicki Deli‐Peri, Luca De Palma, Nabeel Diab, Petia Dimova, Carolina Munoz Castro, Georg Dorfmüller, Jana Domínguez, Gianluca D'Onofrio, Christin Eltze, Johannes M. Nico Enslin, András Fogarasi, Siby Gopinath, Renzo Guerrini, Giulia Nobile, Masaki Iwasaki, Sita Jayalakshmi, Takayuki Kikuchi, Noritsugu Kunihiro, Paweł Kowalczyk, Niklaus Krayenbühl, Pavel Kršek, Thilo Kalbhenn, Kensuke Kawai, Cassia Maniquis, Wim Otte, María Ángeles Pérez‐Jiménez, Juan Carlos Pérez‐Poveda, Catherine Riney, Jonathan Roth, Sabine Rona, Karl Rössler, Olga Teo, Oana Tarta Arsene, Laura Tassi, Hartlieb Till, T S Dhanya, Catalina Soto Órdenes, Piradee Suwanpakdee, Steffen Syrbe, Nigel Peter Symss, Jay Shetty, Dilşad Türkdoğan, Takehiro Uda, Naotaka Usui, Vrajesh Udani, Jithangi Wanigasinghe, Yi Wang

**Affiliations:** ^1^ Department of Neuropediatrics University Children's Hospital, University of Zurich Zurich Switzerland; ^2^ Children's Research Center University Children's Hospital Zurich Zurich Switzerland; ^3^ Neuroscience Center Zurich Eidgenössische Technische Hochschule Zurich and University of Zurich Zurich Switzerland; ^4^ University of Zurich Zurich Switzerland; ^5^ Great Ormond Street Hospital for Children London UK; ^6^ Epilepsy Department Great Ormond Street Hospital for Children, London and Royal Aberdeen Children's Hospital Aberdeen UK; ^7^ Epilepsy Unit Hospital Sant Joan de Déu, member of European Reference Network EpiCARE Barcelona Spain; ^8^ Meyer Children's Hospital Istituto di Ricovero e Cura a Carattere Scientifico and University of Florence Florence Italy; ^9^ Epilepsy Center, Neurological Institute, Cleveland Clinic Cleveland Ohio USA; ^10^ Department of Child Neurology Utrecht Brain Center, University Medical Center Utrecht Utrecht the Netherlands; ^11^ Division of Neurosurgery, Department of Surgery Neuroscience Institute, University of Cape Town Cape Town South Africa; ^12^ Mediclinic Constantiaberg Hospital, Division of Neurology Neuroscience Institute, University of Cape Town Cape Town South Africa; ^13^ Pediatric Epilepsy Center, Peking University First Hospital Peking China; ^14^ Epilepsy Center, Shenzhen Children's Hospital Shenzhen China; ^15^ Department of Neurology Hospital de Pediatría “Prof. Dr. Juan P Garrahan” Buenos Aires Argentina; ^16^ Pediatric Neurosurgery Department Rothschild Foundation Hospital, Centre de Compétence Maladies Rares, Epilepsies Rares, Member of European Reference Network EpiCARE Paris France; ^17^ Department of Epileptology Bethel Epilepsy Center, Medical School Ostwestfalen‐Lippe, Bielefeld University Bielefeld Germany; ^18^ Clinica Cukiert Sao Paolo Brazil; ^19^ Queensland Children's Hospital Brisbane Queensland Australia; ^20^ Department of Neurological Surgery Vanderbilt University Medical Center Nashville Tennessee USA; ^21^ Great Ormond Street Hospital for Children NHS Trust, Developmental Neurosciences University College London–Great Ormond Street Institute of Child Health London UK; ^22^ Department of Neurosurgery David Geffen School of Medicine at University of California Los Angeles California USA; ^23^ Department of Pediatrics Medical University of Vienna, member of European Reference Network EpiCARE Vienna Austria; ^24^ Children's Hospital at Westmead Sydney New South Wales Australia; ^25^ Fundación Hospital Pediátrico la Misericordia Bogotá Colombia; ^26^ Division of Neurology, Department of Pediatrics Centre Hospitalier Universitaire Sainte Justine and Université de Montréal Montreal Quebec Canada; ^27^ Queen Silvia Children's Hospital Sahlgrenska University Gothenburg Sweden; ^28^ Royal Children's Hospital Melbourne Victoria Australia; ^29^ Center for Pediatric Neurology, Neurorehabilitation, and Epileptology Schoen‐Clinic Vogtareuth Germany; ^30^ Gleneagles Hospital Chennai Tamilnadu India; ^31^ Division of Pediatric Neurosurgery, Department of Surgery Texas Children's Hospital Houston Texas USA; ^32^ Department of Neurosurgery Baylor College of Medicine Houston Texas USA; ^33^ Department of Neurology and Epileptology Children's Memorial Health Institute Warsaw Poland; ^34^ Department of Pediatric Neurology Charles University and Motol and Homolka University Hospital, full member of European Reference Network EpiCARE Prague Czech Republic; ^35^ Epilepsy Research Centre Prague–EpiReC Consortium Prague Czech Republic; ^36^ Department of Neurology Nicklaus Children's Hospital Miami Florida USA; ^37^ Deenanath Mangeshkar Hospital and Research Center Pune India; ^38^ Neurology, Epilepsy, and Movement Disorders Unit Bambino Gesù Children's Hospital, IRCCS, full member of European Reference Network EpiCARE Rome Italy; ^39^ Department of Neurology All India Institute of Medical Sciences New Delhi India; ^40^ Bethesda Children's Hospital Budapest Hungary; ^41^ Division of Paediatric Neurology, Department of Paediatrics and Child Health Neuroscience Institute, University of Cape Town and Red Cross War Memorial Children's Hospital Cape Town South Africa; ^42^ Hospital for Sick Children Toronto Ontario Canada; ^43^ Mayo Clinic Rochester Minnesota USA

**Keywords:** developmental and epileptic encephalopathies (DEEs), genetic testing, neuromodulation, pediatric epilepsy surgery, presurgical evaluation, reoperations

## Abstract

**Objective:**

Pediatric epilepsy surgery is well established, but contemporary global data on referral and presurgical evaluation practices are lacking. This International League Against Epilepsy (ILAE) Pediatric Epilepsy Surgery Task Force study provides an updated overview of current trends and regional differences.

**Methods:**

Group‐level data were collected from 61 epilepsy surgery programs (49 pediatric‐only) across 29 countries and six continents, identified through ILAE networks, and included all children and adolescents treated in 2023 who underwent presurgical evaluation/epilepsy surgery.

**Results:**

Group‐level data were available for 2427 patients. Mean age at surgery was 9.1 ± 4.9 years; mean epilepsy duration was 5.3. At surgery, 3.2% were <1 year old (highest in Oceania: 5.1%), and 6.1% were nonpharmacoresistant (highest in Europe: 15.0%). Prior neurosurgery was reported in 14.2% (highest in North America: 28.8%), including 8.0% resections (6.1% for epilepsy, 1.5% for tumors; highest in Oceania: 16.5%), 2.3% disconnections (1.3% corpus callosotomy; highest in South America: 4.7%), and 4.2% neuromodulation (3.7% vagal nerve stimulation, .2% responsive neurostimulation, one deep brain stimulation; highest in North America: 12.2%). Developmental and epileptic encephalopathies (DEEs) at surgery included Lennox–Gastaut syndrome (7.4%), infantile epileptic spasms syndrome (5.1%), and DEE with spike–wave activation in sleep (1.5%). Presurgical investigations included fluorodeoxyglucose positron emission tomography (52.6%; highest in Oceania: 79.7%), genetic testing (46.8%; highest in Asia: 54.3%), magnetic resonance imaging (MRI) postprocessing (32.4%; highest in South America: 53.0%), functional MRI (fMRI; 15.2%; highest in North America: 40.3%), magnetoencephalography (11.9%; highest in North America: 39.3%), single photon emission computed tomography (9.6%; highest in North America: 22.2%), high‐density electroencephalography (EEG; 1.9%; highest in Europe: 4.7%), source localization (1.6%; highest in Oceania: 7.6%), Wada test (1.2%; highest in North America: 3.5%), and EEG‐fMRI (.5%; highest in Europe: 1.1%).

**Significance:**

Despite some early surgeries, including in infancy and before pharmacoresistance, mean epilepsy duration before surgery remains >5 years. Reoperations are common, with resection more frequent than neuromodulation. Genetic testing in nearly half of patients reflects its growing relevance, and the high rate of DEEs underscores the complexity of surgical candidates.

**Key points**

- Group‐level data from 61 epilepsy surgery programs in 29 countries (*n* = 2427) across six continents provide a global overview of pediatric epilepsy surgery practices in 2023.
- Despite some early interventions, including in infancy and before pharmacoresistance, mean epilepsy duration prior to surgery remains >5 years.
- Prior neurosurgery was reported in 14.2% of cases, including 8.0% resections (6.1% for epilepsy, 1.5% for tumors) and 2.3% disconnections.
- Prior neuromodulation was reported in 4.2% of cases, most commonly vagal nerve stimulation.
- Genetic testing was performed in 46.8% of cases, reflecting its growing diagnostic role.
- A substantial proportion of patients had severe developmental and epileptic encephalopathies, including Lennox–Gastaut (7.4%) and infantile epileptic spasms syndrome (5.1%).

## 1 INTRODUCTION

Pediatric epilepsy surgery is an evidence‐based intervention,^1^, ^2^ and is increasingly utilized worldwide.^3^, ^4^, ^5^, ^6^, ^7^, ^8^, ^9^ In 2004, the International League Against Epilepsy (ILAE) conducted the first global survey of pediatric epilepsy surgery practices, published in 2008.^10^ That landmark study reported on 543 patients aged ≤18 years across 20 programs in the United States, Europe, and Australia, and provided detailed data on age at surgery, epilepsy duration prior to surgery, reoperation rates, eligible epilepsy syndromes, and use of presurgical investigations.^10^

In the 2 decades since that initial survey, the field has changed substantially. Access to epilepsy surgery has expanded in high‐income countries^11^ and has become increasingly available in low‐ and middle‐income settings.^6^ Referral volumes have grown, and the surgical population has diversified, including more infants,^12^, ^13^, ^14^ children with developmental and epileptic encephalopathies (DEEs),^15^ and patients with genetic epilepsies.^16^, ^17^ Advances in neuroimaging^18^ and genetic diagnostics^19^ have improved candidate selection, and minimally invasive approaches such as laser interstitial thermal therapy and novel neuromodulation techniques^4^ such as responsive neurostimulation (RNS) and deep brain stimulation (DBS) have broadened the therapeutic spectrum.

Updated 2022 ILAE recommendations^20^ emphasize early presurgical evaluation—ideally as soon as pharmacoresistance is established—and highlight the importance of timely referral, including for infants and selected nonpharmacoresistant patients with identifiable lesions in noneloquent cortex. As clinical pathways become more individualized, reassessment of referral and presurgical evaluation practices is warranted. Although recent studies have reported national, regional, single‐center, or multicenter trends,^3^, ^7^, ^11^, ^21^ none has provided a comprehensive global perspective.

To address this gap, the ILAE Pediatric Epilepsy Surgery Task Force within the Surgical Therapies Commission 2021–2025 launched an international initiative to update and expand the 2004 survey.^10^ This study systematically captured center‐level referral patterns and presurgical evaluation strategies, invasive explorations, surgical procedures, and etiologies across a broad, globally representative sample of epilepsy surgery programs. Here, we report referral patterns and presurgical evaluation strategies; other domains are presented separately. By documenting changes over the past 2 decades and highlighting regional variation, we aimed to provide updated benchmarks and inform clinical guidance and research priorities in pediatric epilepsy surgery.

## 2 MATERIALS AND METHODS

### 2.1 Study design

This international, cross‐sectional survey collected group‐level data on referral patterns and presurgical evaluation practices in pediatric epilepsy surgery. Eligible patients were aged ≤18 years and underwent epilepsy surgery in 2023. Included procedures were resective, disconnective, ablative, or neuromodulatory, as well as invasive electroencephalographic (EEG) evaluations, including cases where no subsequent intervention was performed. Patients who underwent noninvasive presurgical evaluation but did not proceed to invasive EEG evaluation or surgery were excluded. Postsurgical seizure, developmental, or behavioral outcomes were not assessed.

### 2.2 Survey development and scope

A web‐based data entry form was developed in REDCap and hosted on secure servers at the University Children's Hospital Zurich. The form was designed to collect standardized, deidentified group‐level clinical data on pediatric epilepsy surgery cases treated in 2023.

### 2.3 Participating centers and inclusion criteria

To achieve broader global coverage than the 2004 ILAE survey,^10^ which included 20 programs in Europe, the United States, and Australia, epilepsy surgery centers from all six ILAE regions (Africa, Asia‐Oceania, Eastern Mediterranean, Europe, Latin America, and North America) were invited to participate. The initial call was disseminated through members of the ILAE Pediatric Epilepsy Surgery Task Force, the ILAE Surgical Therapies Commission, and regional ILAE leadership. In addition, centers that had participated in the 2004 ILAE survey were contacted directly. A formal record of invited but nonparticipating centers was not maintained. Eligible centers included both pediatric‐only and mixed‐age programs with dedicated pediatric services. Participation required the ability to provide group‐level data on all pediatric patients (≤18 years) who underwent surgery or invasive EEG in 2023. Centers were excluded if their pediatric surgical activity was largely limited to vagal nerve stimulator (VNS) implantation, as these programs were not considered to reflect the scope of pediatric epilepsy surgery practice addressed in this survey.^22^

### 2.4 Data collection

Each center appointed one or more local leads responsible for data extraction and entry into REDCap. The survey did not capture the specific professional role of local leads. Data collection covered referral characteristics (e.g., age at seizure onset, age at surgery, epilepsy syndrome, pharmacoresistance, prior neurosurgery) and the use of presurgical investigations including video‐EEG, structural magnetic resonance imaging (MRI), neuropsychological testing, fluorodeoxyglucose positron emission tomography (FDG‐PET), single photon emission computed tomography (SPECT), high‐density EEG, magnetoencephalography (MEG), EEG source localization, EEG–functional MRI (EEG‐fMRI), MRI postprocessing, functional MRI, Wada test, and genetic testing. The survey documented their use and frequency but not their impact on surgical decision‐making.

### 2.5 Statistics

Descriptive statistics were calculated for all study variables. Categorical variables are reported as frequencies and percentages, and continuous variables as means and SDs.

Comparisons across continents were assessed using generalized estimating equations (GEE). For continuous outcomes, a Gaussian family with identity link was used; for proportional outcomes, a binomial family with a logit link was applied. Models were fitted using the *geeglm()* function from the geepack package in R. To account for clustering within countries, an exchangeable correlation structure was used. To mitigate center‐size imbalance without allowing a small number of hubs to dominate the analysis, GEE models incorporated weights proportional to each center's patient count. Europe served as the reference category for all continent‐level comparisons, because it included a large proportion of participating centers and patients and provided a stable comparator for analysis. Africa was excluded from inferential analyses due to small sample size (*n* = 17).

For outcomes with few events, estimates were imprecise (wide confidence intervals) and should be interpreted cautiously. When zero or sparse counts yielded unstable GEE estimates, results were considered not estimable; these are provided in the Supporting Tables but are not discussed in the main text.

A separate GEE model assessed the association between the proportion of patients with prior surgery and mean epilepsy duration across centers.

Statistical significance was defined as a two‐sided *p*‐value ≤ .05. All analyses were performed using R (v. 4.3.1).^23^

## 3 RESULTS

### 3.1 Participating centers and patient cohort

**FIGURE 1:**
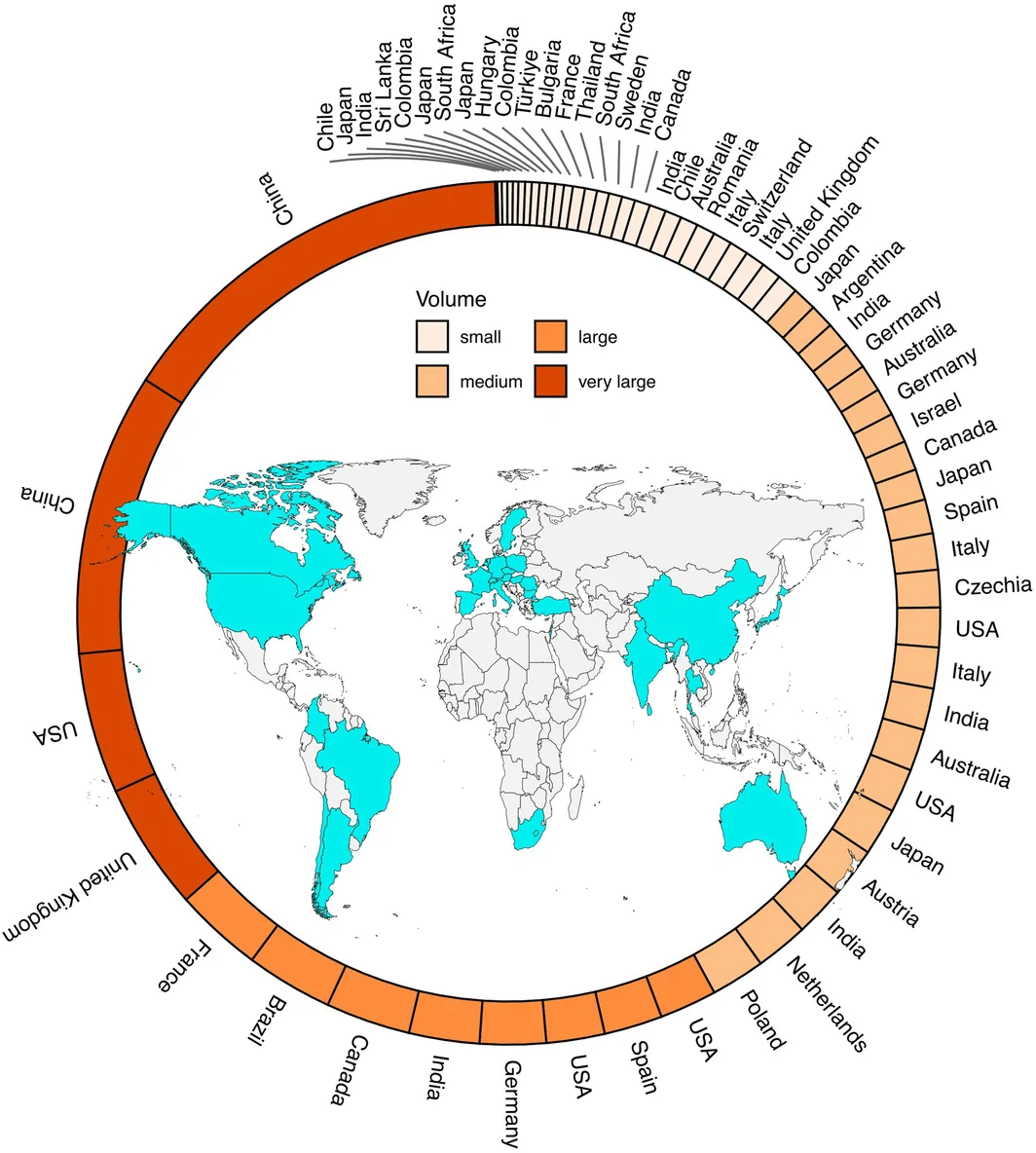
Global distribution of contributing epilepsy surgery programs. Data were contributed by 29 countries across six continents (highlighted in cyan), representing a total of 2427 pediatric patients who underwent epilepsy surgery in 2023. The central map illustrates the geographic spread of participating countries. The surrounding ring is subdivided into segments corresponding to individual centers. Segment width is proportional to each center's patient count, and segment color indicates surgical volume: small (<20 patients, beige), medium (20–49, light orange), large (50–99, orange), and very large (≥100, dark orange).

We collected data on 2427 children and adolescents who underwent epilepsy surgery in 2023 from 61 epilepsy surgery programs in 29 countries across six continents (Figure 1, Table S1). Of these, 49 centers were pediatric‐only and 12 were mixed‐age centers with dedicated pediatric services. Based on World Bank income classification,^24^ 42 of 61 participating centers (68.9%) were located in high‐income countries and 19 (31.1%) in low‐ or middle‐income countries. Patient distribution by continent was as follows: Asia (*n* = 960), Europe (*n* = 795), North America (*n* = 427), South America (*n* = 149), Oceania (*n* = 79), and Africa (*n* = 17). Annual surgical volume varied; 27 centers treated fewer than 20 patients (small volume), 22 treated 20–49 patients (medium), eight treated 50–99 patients (large), and four treated 100 or more (very large). The median number of patients per center was 24 (interquartile range = 12–45). The cohort comprised 55.3% males and 44.7% females. The proportion of females varied across regions, ranging from 40.3% in Asia to 57.0% in South America, and was below 50% in Asia and Europe.

### 3.2 Referral patterns

**FIGURE 2:**
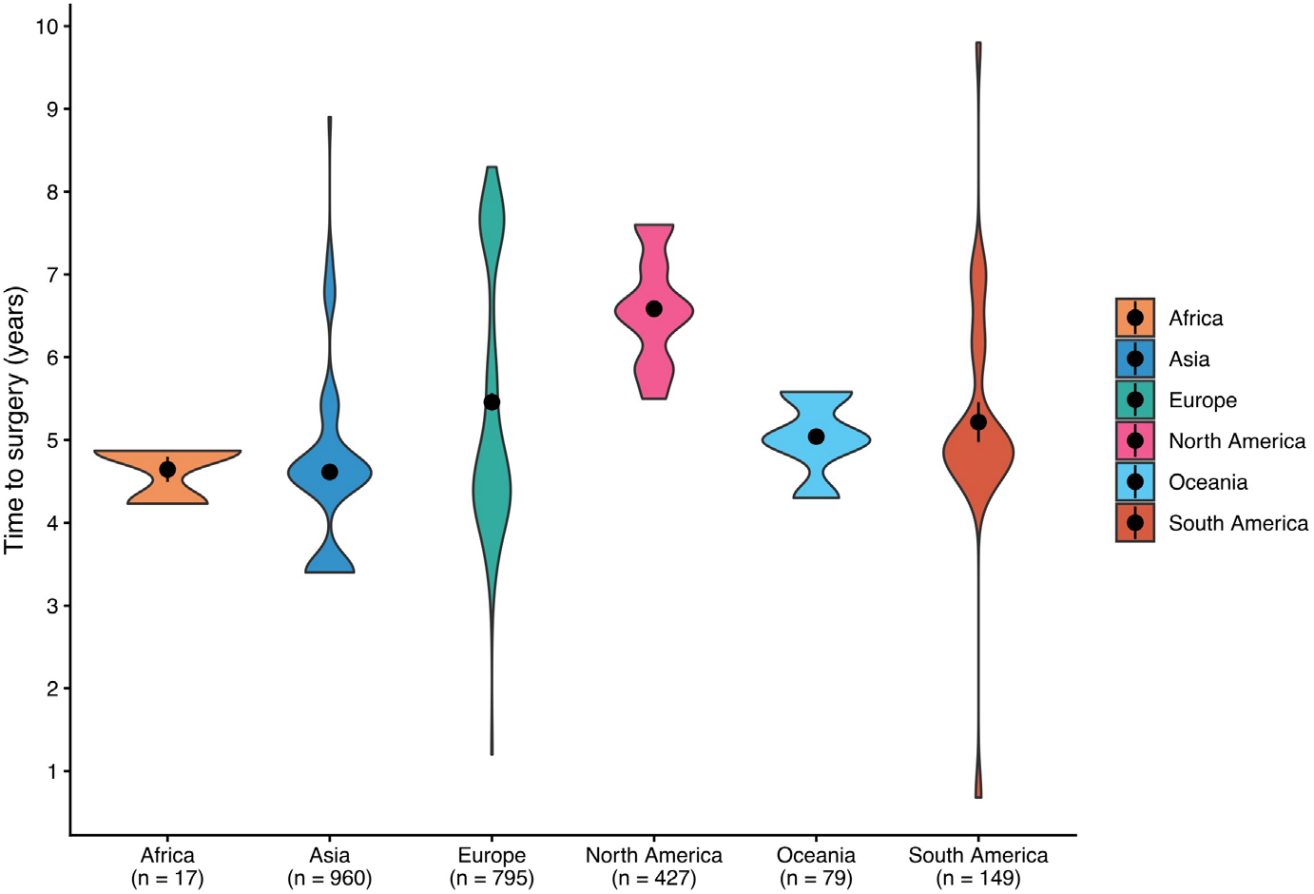
Time from epilepsy onset to surgery by continent. Violin plots show the distribution of time from epilepsy onset to surgery by continent. Each violin represents the distribution density for one continent; the black dot indicates the mean. Across the cohort, the mean duration from epilepsy onset to surgery was 5.3 years. Asia had the shortest interval (4.6 years) and North America the longest (6.6 years). Compared with Europe, patients in North America had a significantly longer duration from epilepsy onset to surgery (*p* = .027).

The mean epilepsy duration prior to surgery was 5.3 years, ranging from 4.6 years in Asia to 6.6 years in North America. Compared to Europe, epilepsy duration was significantly longer in North America (GEE, *p* = .027; Figure 2, Table S2). The mean age at seizure onset was 3.8 ± 3.8 years, lowest in Asia (3.4 years) and highest in South America (5.1 years), although no regional differences were statistically significant compared to Europe. The mean age at surgery was 9.1 ± 4.9 years, ranging from 8.0 years in Asia to 10.7 years in North America. Compared to Europe, patients in North America underwent surgery at a significantly older age (*p* = .031), whereas patients in Asia underwent surgery at a significantly younger age (*p* = .039).

**FIGURE 3:**
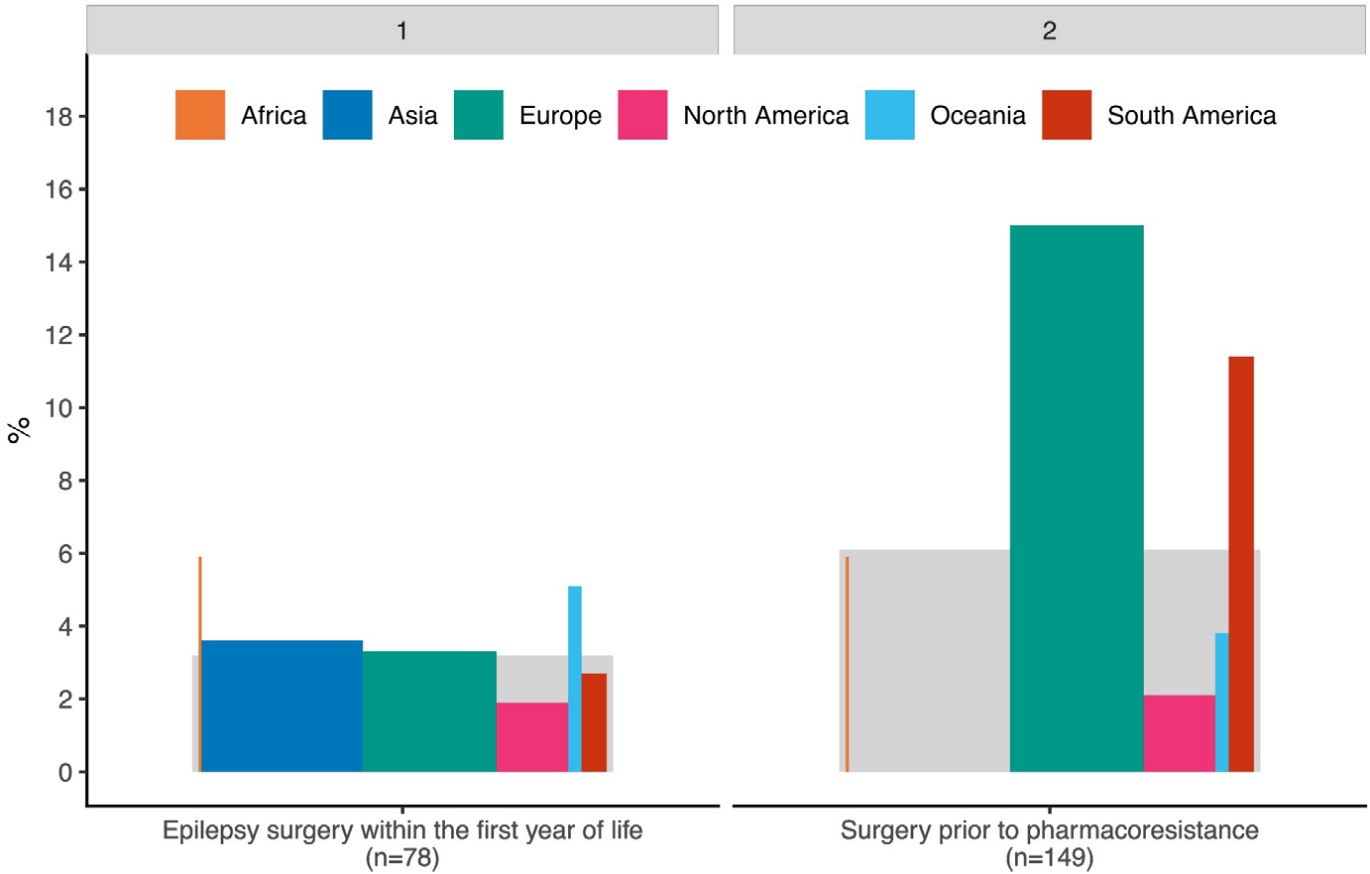
Surgery in infancy and surgery before pharmacoresistance by continent. Bar plots show the proportion of patients who underwent epilepsy surgery (1) in the first year of life and (2) before meeting pharmacoresistance criteria. Globally, surgery in infancy was reported in 3.2% of patients and surgery before pharmacoresistance in 6.1%. Surgery in infancy was more frequent in Oceania (5.1%), and surgery before pharmacoresistance was more common in Europe (15.0%). Bar width reflects regional sample size; gray background bars indicate global percentages.

Surgery in the first year of life was reported in 3.2% of all patients, with the highest rates in Oceania (5.1%) and lowest in North America (2%). However, no region differed significantly from Europe. Surgery before meeting pharmacoresistance criteria was reported in 6.1% of all patients, most commonly in Europe (15.0%) and not at all in Asia. Compared to Europe, North America had significantly lower odds of surgery before meeting pharmacoresistance criteria (*p* < .001; Figure 3, Table S2).

### 3.3 Surgical history prior to current procedure

**FIGURE 4:**
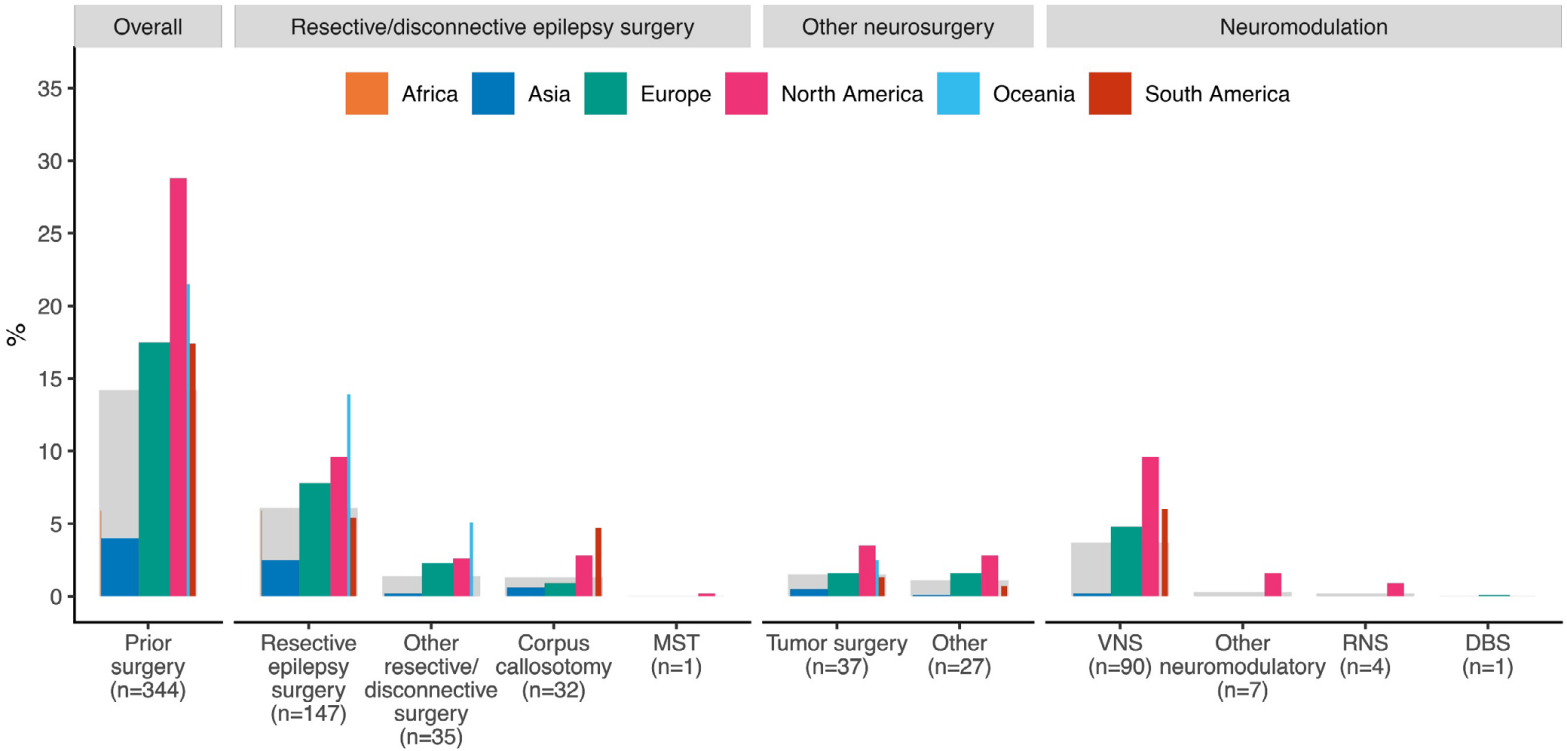
Prior neurosurgery by continent. Bar plots show the proportion of patients with prior neurosurgical procedures, grouped as resective/disconnective epilepsy surgery, other neurosurgery, and neuromodulation. Globally, 14.2% of patients had prior neurosurgery, ranging from 4.0% in Asia to 28.8% in North America. Prior resective/disconnective surgery included prior epilepsy surgery in 6.1% of patients and corpus callosotomy in 1.3%. Other neurosurgery included tumor resections in 1.5% of patients. Neuromodulation preceded surgery in 4.2% overall, most commonly vagal nerve stimulation (VNS; 3.7%), followed by responsive neurostimulation (RNS; .2%) and deep brain stimulation in one case (DBS; .04%). Prior neuromodulation was more common in North America (12.2%) and absent in Oceania. Bar width reflects regional sample size; gray background bars indicate global percentages. MST, multiple subpial transections.

Prior neurosurgery, including prior epilepsy surgery and neurosurgery for other indications, was reported in 14.2% of all patients, ranging from 4.0% in Asia to 28.8% in North America. Compared to Europe, prior surgery was significantly more frequent in North America (GEE, *p* = .029; Figure 4, Table S3) and less frequent in Asia (*p* = .003).

Prior resective procedures had been performed in 8.0% of all patients (6.1% for epilepsy and 1.5% for tumor), ranging from 3.0% in Asia to 16.5% in Oceania. Prior disconnections were not significantly different when compared to Europe. Disconnective procedures were reported in 2.3% of all patients, including 1.3% with prior corpus callosotomy and one case of multiple subpial transections. These were most common in Oceania (5.1%) and least common in Asia (.7%), but differences were not significant compared to Europe; estimates for Asia could not be calculated due to sparse events.

Neuromodulation prior to the index epilepsy surgery (resective, disconnective, or ablative) was reported in 4.2% of all patients: 3.7% VNS, .2% RNS, and one case of DBS. Rates of prior neuromodulation were highest in North America (12.2%), with no cases reported in Oceania. Compared to Europe, prior neuromodulation was significantly less common in Asia (*p* = .007).

A higher proportion of patients with prior neurosurgery was significantly associated with longer epilepsy duration (*p* < .001; Table S4). Specifically, a 10% increase in the proportion of patients with prior neurosurgery was associated with an estimated 6‐month‐longer epilepsy duration at the time of index surgery.

### 3.4 Epilepsy syndromes

A prior history of an electroclinical syndrome was reported in 24.8% of all patients, ranging from 15.3% in Asia to 37.6% in South America. The most common were infantile epileptic spasms syndrome (IESS; 12.3%), Lennox–Gastaut syndrome (LGS; 6.2%), and developmental epileptic encephalopathy with spike‐and‐wave activation in sleep (DEE‐SWAS; 1.6%). Compared to Europe, a history of LGS was significantly more common in South America (GEE, *p* < .001), whereas DEE‐SWAS was significantly less common in North America (*p* = .001) and South America (*p* = .007), and no DEE‐SWAS cases were reported in Oceania (Figure S1, Table S5).

**FIGURE 5:**
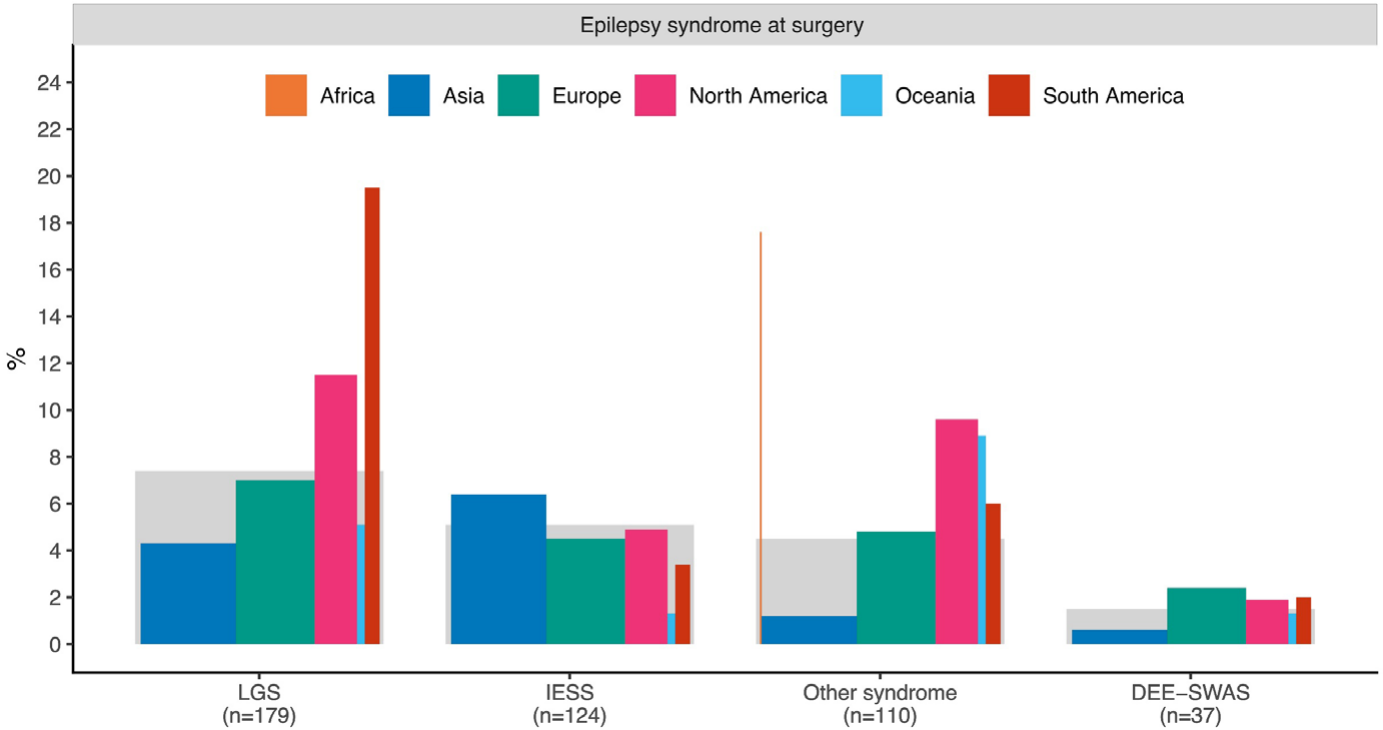
Epilepsy syndromes at presurgical evaluation by continent. Bar plots show the proportion of patients diagnosed with infantile epileptic spasms syndrome (IESS), Lennox–Gastaut syndrome (LGS), developmental epileptic encephalopathy with spike‐and‐wave activation in sleep (DEE‐SWAS), or other defined epilepsy syndromes at presurgical evaluation. Globally, 18.5% of patients presented with an epilepsy syndrome, most commonly LGS (7.4%), IESS (5.1%), and DEE‐SWAS (1.5%). LGS (19.5%) was most frequent in South America, IESS in Asia (6.4%), and DEE‐SWAS in Europe (2.4%). Bar width reflects regional sample size; gray background bars indicate global percentages.

At the time of presurgical evaluation, a current electroclinical syndrome was identified in 18.5% of all patients, ranging from 12.5% in Asia to 30.9% in South America. LGS remained the most frequent (7.4%), followed by IESS (5.1%), and DEE‐SWAS (1.5%). Compared with Europe, no significant regional differences were found for IESS, LGS, or DEE‐SWAS (Figure 5, Table S6). At the time of evaluation, IESS was most common in Asia (6.4%), whereas LGS (19.5%) was most common in South America and DEE‐SWAS (2.4%) was most frequent in Europe.

### 3.5 Diagnostic modalities

**FIGURE 6:**
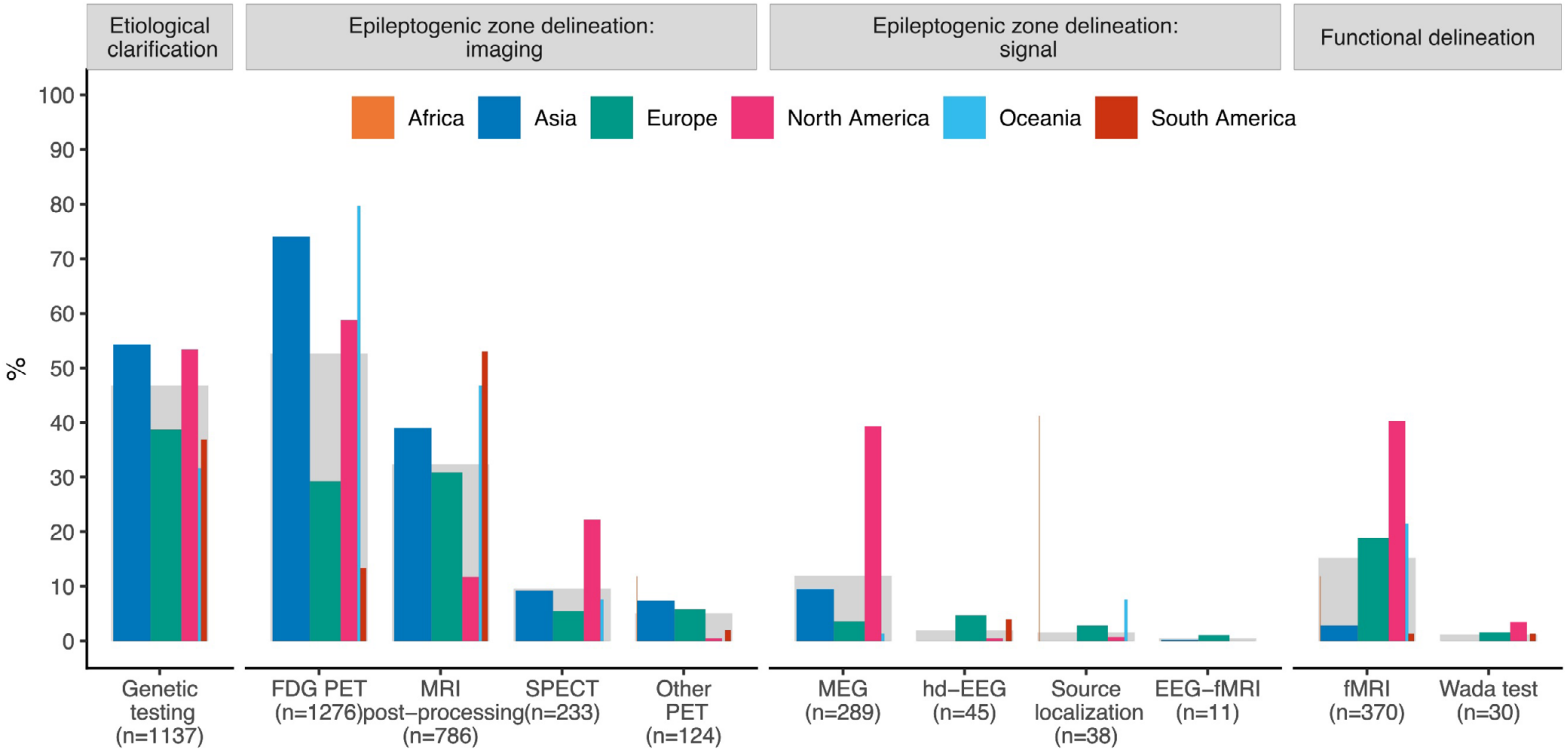
Auxiliary presurgical investigations by continent. Bar plots show auxiliary presurgical investigations, grouped by purpose: (1) etiological clarification, (2) epileptogenic zone delineation by imaging or electrophysiology, and (3) functional mapping. The most frequently used modalities globally were fluorodeoxyglucose positron emission tomography (FDG PET; 52.6%), genetic testing (46.8%), magnetic resonance imaging (MRI) postprocessing (32.4%), functional MRI (fMRI; 15.2%), magnetoencephalography (MEG; 11.9%), and single photon emission computed tomography (SPECT; 9.6%). Other techniques—including the Wada test, high‐density electroencephalography (hd‐EEG), source localization, EEG‐fMRI, and non‐FDG PET—were each used in <2.0% of the cohort, with absent or minimal use in several regions. Bar width is proportional to the number of patients per region. Gray background bars indicate global usage rates.

Core presurgical investigations—structural MRI, long‐term video‐EEG, and neuropsychological evaluation—were performed in 98.8%, 92.7%, and 81.0% of all patients, respectively. The mean number of diagnostic tests per patient was 4.5, with the highest mean reported in North America (4.9), Asia (4.8), and Oceania (4.8), followed by Europe (4.1) and South America (3.8). The number of diagnostic modalities used in Asia was significantly higher compared to Europe (*p* = .042, GEE; Figure 6, Table S7).

For epileptogenic zone localization, FDG‐PET was the most commonly used advanced imaging modality beyond MRI, applied in 52.6% of all patients. Utilization ranged from 13.4% in South America to 79.7% in Oceania. Compared to Europe, FDG‐PET use was significantly more frequent in Asia (*p* < .001), Oceania (*p* = .001), and North America (*p* = .003), and significantly less frequent in South America (*p* = .031; Figure 6, Table S8). MRI postprocessing was performed in 32.4% of cases, ranging from 11.7% in North America to 53.0% in South America. No significant differences were observed compared to Europe. SPECT was applied in 9.6% of cases, most commonly in North America (22.2%) and not at all in South America. Compared to Europe, SPECT use was significantly more common in North America (*p* = .005).

For functional mapping, fMRI was applied in 15.2% of all patients, ranging from 1.3% in South America to 40.3% in North America. Compared to Europe, fMRI use was significantly higher in North America (*p* = .002) and significantly lower in Asia (*p* < .001) and South America (*p* = .016). The Wada test was used in 1.2% of all patients, most frequently in North America (3.5%) and not at all in Asia or Oceania.

Among signal analysis techniques, MEG was used in 11.9% of all patients overall, with the highest use in North America (39.3%) and no use in South America. Compared to Europe, MEG use was significantly higher in North America (*p* < .001). High‐density EEG was used in 1.9% of all patients, most commonly in Europe (4.7%), with no reported use in Asia or Oceania. Source localization was applied in 1.6% of all patients, most frequently in Oceania (7.6%), with no reported use in Asia or South America. EEG‐fMRI was performed in .5% of cases, with the highest rate in Europe (1.1%) and no use in Oceania, North America, or South America.

Genetic testing was the second most commonly used auxiliary investigation, performed in 46.8% of all patients. Utilization ranged from 31.6% in Oceania to 54.3% in Asia, with no significant regional differences compared to Europe.

## 4 DISCUSSION

This international survey provides the most comprehensive global overview to date of pediatric epilepsy surgery practices, capturing referral characteristics and presurgical evaluations from 61 programs across 29 countries. It builds on the landmark 2004 ILAE survey,^10^ which included 543 patients from 20 centers across North America, Europe, and Oceania. In contrast, the present dataset comprises group‐level data on 2427 patients—almost five times as many—and includes all six ILAE regions, incorporating previously underrepresented areas such as Asia, South America, and Africa. This broader geographic scope likely reflects both expansion of pediatric epilepsy surgery capacity over the past 2 decades, including in high‐ and middle‐income countries, and more systematic international outreach through current ILAE networks. Notably, Africa was represented by only two centers within a single city in one country and contributed the fewest patients overall, leading to exclusion from several statistical analyses. This underrepresentation underscores the need to expand epilepsy surgery capacity and research participation across Africa to improve access and strengthen global evidence.

### 4.1 Persistent delays in surgical referral

Despite growth in surgical programs and patient volume, delays persist. The mean epilepsy duration prior to surgery was 5.3 years, similar to the 6.3 years reported in the 2004 ILAE survey.^10^ This finding indicates ongoing prolonged exposure to uncontrolled seizures before referral,^25^ despite accumulating evidence supporting earlier intervention.^25^ The ILAE Pediatric Epilepsy Surgery Task Force highlighted the importance of timely referral nearly 2 decades ago,^26^ and multiple studies have since reinforced that earlier evaluation and surgery improve both seizure and cognitive outcomes.^27^, ^28^ Yet, surgery remains underutilized; US data suggest that fewer than one third of eligible children ultimately receive surgery, with slower growth and longer delays among socially disadvantaged groups, underscoring persistent inequities.^29^ Geographic and systems variability, limited specialist access, and insurance complexity further impede timely referral and evaluation, even in high‐income settings with established programs.^30^, ^31^ Practical steps include earlier referral protocols, rapid‐access or telemedicine‐based presurgical consultations to reduce distance and waiting time, and standardized shared decision‐making with proactive outreach to primary neurologists and families to normalize earlier evaluation.^32^, ^33^

Surgery in the first year of life was reported in 3.2% of all patients, modestly higher than the 1.5% reported in the 2004 ILAE survey, but still low given the high incidence of seizure onset in infancy^10^ and growing evidence that infants with focal lesions benefit from early surgery.^12^, ^13^, ^14^, ^28^, ^34^ Many such cases are DEEs, where early seizure control may mitigate the neurodevelopmental impact of pharmacoresistant epilepsy. As epilepsy surgery is safe and effective across the lifespan,^12^, ^13^, ^14^ early access should be prioritized. Epilepsy duration remains the only modifiable predictor of surgical outcome,^35^, ^36^, ^37^, ^38^ and early intervention, especially in infancy, may leverage critical windows of neuroplasticity, such as those supporting language development, and enable optimal long‐term cognitive and behavioral outcomes.

Although pharmacoresistance remains the main threshold for presurgical evaluation, surgery before meeting formal criteria^39^ was reported in 6.1% of all patients, suggesting that early referral is increasingly considered in selected cases. This finding adds important new information, as previous large surveys—including the 2004 ILAE survey and several regional series—either did not assess this variable^3^, ^5^, ^9^, ^10^ or only included pharmacoresistant cases by design.^4^, ^40^ Although still debated, early surgery in nonpharmacoresistant patients with clear structural lesions^28^, ^41^ has been associated with favorable outcomes, including seizure freedom and antiseizure medication (ASM) withdrawal.^42^, ^43^

Current ILAE recommendations support early referral once pharmacoresistance is established and endorse surgical consideration in selected nonpharmacoresistant patients with lesions in noneloquent cortex.^20^ Several lines of evidence support earlier surgical intervention. In children with early onset epilepsy, particularly those with focal cortical dysplasia,^44^ the likelihood of sustained response to ASM is low, and failure of even one ASM significantly increases the risk of pharmacoresistance.^39^ Longer epilepsy duration is consistently associated with poorer postsurgical seizure outcomes, regardless of age, localization, or etiology.^45^, ^46^, ^47^ Early surgery may also facilitate developmental gains, especially if ASM can be reduced or withdrawn.^48^, ^49^, ^50^, ^51^, ^52^ Conversely, delayed surgery risks cognitive decline due to ongoing seizures, interictal discharges, or ASM side effects,^48^, ^50^ and may miss critical windows for neuroplasticity, limiting the potential for functional recovery.

### 4.2 Prior neurosurgical interventions and treatment sequencing

Prior neurosurgical interventions were reported in 14.2% of cases, less frequent than in the 2004 ILAE survey (22.0%).^10^ Most reported prior procedures were resective, with smaller proportions involving tumors (1.5%) or disconnections (2.3%). The lower reoperation rate may reflect changes in case mix (more infants, DEEs, and MRI‐negative cases)^15^; greater use of staged treatment strategies, often starting with less invasive interventions; more definitive primary resections enabled by improved planning^53^; and inclusion of regions not represented in the earlier survey where constraints may limit multiple interventions. Still, reoperations remained more common in some settings, particularly North America, where nearly one third of patients had prior neurosurgery. These patterns may reflect regional differences in referral pathways and sequencing of less invasive interventions ahead of more extensive procedures aimed at seizure freedom.^54^

In contrast to the earlier survey, where tumors accounted for 40% of reoperations,^10^ tumor‐related prior resections represented 1.5% of all patients in this cohort. Although denominators differ, precluding direct comparison, this may reflect more targeted initial treatment of low‐grade epilepsy‐associated tumors (LEATs).^53^ Optimal management of LEATs requires multidisciplinary input from epileptology, neuro‐oncology, and neurosurgery, and surgical planning should address both the tumor and the surrounding epileptogenic cortex to optimize seizure control, neurological function, and oncologic outcome.^55^

Prior neuromodulation was reported in 4.2% of all patients, predominantly VNS, similar to the 5.0% reported in the 2004 ILAE survey, which was conducted before wider use of other devices such as RNS or DBS. Pediatric labeling varies by region and device, and these modalities are used off‐label in select centers. Regional variation was notable; in North America, 12.2% received neuromodulation before surgery, suggesting that some programs prioritize seizure reduction‐oriented neuromodulation ahead of resection, which may postpone seizure freedom‐oriented surgery in candidates with realistic chances of seizure freedom. This pattern mirrors the 2004 survey, where VNS use was more common in US centers, indicating that the practice has persisted. Children with pharmacoresistant epilepsy should be evaluated at expert comprehensive epilepsy centers, and when they are reasonable candidates for seizure freedom‐oriented surgery, this should be prioritized over neuromodulation, which more often offers seizure reduction than seizure freedom.

### 4.3 Surgical candidacy for DEEs

In our cohort, 7.4% of patients were diagnosed with LGS and 5.1% with IESS, similar to the 2004 ILAE survey (8.6% and 4.0%, respectively).^10^ This underscores the ongoing inclusion of complex electroclinical phenotypes in pediatric epilepsy surgery and growing recognition that selected patients can benefit from surgery even with bilateral or multifocal discharges, when a focal or lateralized epileptogenic zone or lesion is identified.^12^, ^28^, ^34^ Selected patients with IESS can achieve excellent seizure and developmental outcomes with early surgery, particularly when a clear lesion is present.^12^, ^34^, ^56^ However, many are referred only after initial response to hormonal therapy and later relapse.^57^ The higher rate of LGS may reflect the broader range of surgical strategies now available—resective, disconnective, and neuromodulatory—tailored to etiology.^15^ These findings underline the need for referral to specialized pediatric centers with expertise in early onset epilepsies and DEEs, where individualized treatment decisions are made through multidisciplinary evaluation.^22^, ^26^

Genetic testing was performed in 46.8% of patients, reflecting its growing role in presurgical evaluation. In younger children, particularly those with early seizure onset, developmental delay, MRI‐negative findings, or suggestive family history, genetic results increasingly inform candidacy, including in DEEs.^15^ Current recommendations endorse early, individualized etiologic workup in these subgroups to guide management.^58^, ^59^ Notably, genetic testing was absent from the 2006 ILAE presurgical evaluation guidelines^26^ and from both the 2004 global survey^10^ and the 2014 diagnostic test survey.^60^ Since then, genetic testing has become routine in many programs,^61^ driven by advances in epilepsy genomics^62^ and the shift toward precision medicine. Findings may support surgery (e.g., mTOR pathway and *SLC35A2* gene variants^62^, ^63^) or argue against resection when specific pathogenic variants predict poor outcomes (e.g., *SCN8A*, *GABRA1*, *PCDH19*). A confirmed diagnosis may determine whether to proceed with invasive valuation, modify the surgical plan, or defer surgery. In some cases, post hoc tissue testing after invasive evaluation or resection^62^, ^64^, ^65^ clarifies etiology, explains suboptimal outcomes, and guides future counseling.

### 4.4 Evolution in diagnostic modalities

Although core presurgical investigations—video‐EEG, structural MRI, and neuropsychological testing^66^—remained nearly universal (≥81.0%), patients underwent a mean of 4.5 diagnostic tests, reflecting increasingly complex and individualized evaluations, particularly in high‐resource settings such as North America.

Compared with the 2004 ILAE survey,^10^ Wada testing declined sharply (from 9.0% to 1.2%) and fMRI use increased modestly (12.0% to 15.2%), consistent with a broader shift toward noninvasive functional mapping. FDG‐PET use more than doubled (24.0% to 52.6%), possibly reflecting improved spatial resolution, wider availability, and ease of interictal acquisition.^67^ By contrast, SPECT use dropped from 25.0% to 9.6%, possibly related to logistical complexity and less consistent reliability. MEG use quadrupled (3.0% to 11.9%), and techniques absent from both the 2004 ILAE survey^10^ and the 2014 diagnostic test review^60^ such as source localization,^68^, ^69^ high‐density EEG,^70^ and EEG‐fMRI^71^ are now used selectively. Together, these changes reflect technological progress and a shift toward higher resolution, noninvasive strategies.

Nevertheless, considerable regional variation persists. North America reported the highest mean number of tests per patient and more frequent use of MEG, SPECT, and fMRI. MRI postprocessing was more common in South America and less used in North America. PET was widely used in Asia, Oceania, and North America, whereas SPECT and fMRI were rarely used in South America. These differences likely reflect disparities in infrastructure, expertise, and resource availability rather than shared regional or patient‐specific practice frameworks.

Overall, the number and diversity of presurgical investigations have expanded since 2004.^10^ Several modalities, including MRI postprocessing and source localization, have transitioned from research tools into selective clinical use, particularly in high‐resource regions. Whether this broader diagnostic toolkit improves surgical planning or outcomes remains unknown and warrants further study.

### 4.5 Future directions

This study has limitations. Data were collected at the group level, precluding patient‐level analyses and adjustment for individual confounders, and postsurgical outcomes were not assessed. In addition, the absence of a formal record of invited but nonparticipating centers limits assessment of response rates and representativeness. Future studies should use prospective patient‐level data and more systematic center recruitment to evaluate outcomes and the impact of specific presurgical strategies on surgical decision‐making.

## 5 CONCLUSIONS

This global survey provides a contemporary overview of pediatric epilepsy surgery across all ILAE regions. Compared with 2 decades ago, surgical programs are more widely available geographically and diagnostic approaches have diversified, with increased use of advanced imaging and genetic testing. Yet the mean epilepsy duration prior to surgery remains >5 years, and early surgery, especially in infancy or before pharmacoresistance, remains uncommon. DEEs, such as LGS and IESS, continue to comprise a substantial share of surgical candidates. Marked regional differences in diagnostic strategies, reoperation rates, and treatment sequencing indicate limited harmonization of practice. High‐resource centers often use more presurgical tests, but the added clinical value of broader diagnostic workup is uncertain. Clarifying which modalities meaningfully inform surgical planning and outcomes is needed. By capturing center‐level practices across a large global cohort, this study provides updated benchmarks and highlights areas where greater harmonization within regions and patient subgroups may be achievable. Future studies using individual patient data are needed to determine how specific evaluation strategies affect outcomes and resource use.

## AUTHOR CONTRIBUTIONS

Georgia Ramantani, J. Helen Cross, Dorottya Cserpan, Dario Englot, Martha Feucht, Sarah Ferrand Sorbets, Manjari Tripathi, Howard Weiner, Mary Lou Smith, and Elaine Wirrell contributed to the conception and design of the study. All authors contributed data for the article. Georgia Ramantani and Dorottya Cserpan analyzed the data, drafted the manuscript, and prepared the figures, with significant contributions from J. Helen Cross, Mary Lou Smith, and Elaine Wirrell. All authors reviewed, edited, and approved the final version of the manuscript.

## FUNDING INFORMATION

This work was supported by Swiss National Science Foundation (grant 208184; project number 10008856) and the Anna Mueller Grocholski Foundation (to G.R.). The funders had no role in the design or analysis of the study.

## CONFLICT OF INTEREST STATEMENT

The authors declare no conflicts of interest. We confirm that we have read the Journal's position on issues involved in ethical publication and affirm that this report is consistent with those guidelines.

## ETHICS STATEMENT

The collection and analysis of patient data were approved by and performed according to the guidelines and regulations of the local ethics committee (KEK‐ZH Req‐2024‐00058).

## Supporting information


**FIGURE S1.** History of epilepsy syndromes by continent. Bar plots show the proportion of patients with a documented history of infantile epileptic spasms syndrome (IESS), Lennox–Gastaut syndrome (LGS), developmental epileptic encephalopathy with spike‐and‐wave activation in sleep (DEE‐SWAS), or other defined epilepsy syndromes. Globally, 24.8% of patients had a history of an epilepsy syndrome. IESS was the most frequently reported historical syndrome (12.3%), followed by LGS (6.2%) and DEE‐SWAS (1.6%). Compared with Europe, a history of LGS was more common in South America, whereas DEE‐SWAS was less frequent in Oceania, North America, and South America. No significant regional differences were observed for IESS. Bar width is proportional to the number of patients per region. Gray background bars indicate global usage rates.
**TABLE S1.** Pediatric epilepsy surgery centers and investigators participating in the 2023 International League Against Epilepsy survey.
**TABLE S2.** Referral metrics by continent. Generalized estimating equation results comparing continents with Europe are shown. For continuous outcomes (age at epilepsy onset, age at surgery, epilepsy duration), entries represent estimated differences in years with 95% confidence intervals (CIs); negative values indicate a lower mean than Europe, positive values a higher mean. For proportional outcomes (surgery before pharmacoresistance; surgery in the first year of life), entries are odds ratios (ORs) with 95% CIs; OR < 1 indicates a lower rate and OR > 1 a higher rate than Europe. The intercept represents Europe's mean (continuous outcomes) or baseline odds (proportional outcomes). Statistically significant values (*p* < .05) are shown in bold. Africa was excluded due to small sample size (*n* = 17).
**TABLE S3.** Prior neurosurgery by continent. Generalized estimating equation odds ratios with 95% confidence intervals for prior surgery, prior resection, prior disconnection, and prior neuromodulation are shown, comparing each continent with Europe. The intercept represents Europe's baseline odds. Statistically significant values (*p* < .05) are shown in bold. Africa was excluded due to the small sample size (*n* = 17).
**TABLE S4.** Association between reoperation rate and epilepsy duration. Generalized estimating equation estimates describe the relationship between center‐level reoperation rate and mean epilepsy duration (in years). The positive coefficient indicates that higher reoperation rates are associated with longer epilepsy duration. Each 1% increase in reoperation rate corresponds to .05 years (~.66 months) longer duration; a 10% increase corresponds to ~6.6 months longer duration (*p* < .001).
**TABLE S5.** History of syndromes. Generalized estimating equation odds ratios (ORs) with 95% confidence intervals are shown for documented history of infantile epileptic spasms syndrome (IESS), developmental and epileptic encephalopathy with spike‐and‐wave activation in sleep (DEE‐SWAS), and Lennox–Gastaut syndrome (LGS), comparing each continent with Europe. OR < 1 indicates a lower rate and OR > 1 a higher rate than Europe. The intercept reflects Europe's baseline odds. Statistically significant findings (*p* < .05) are shown in bold. Africa was excluded due to the small sample size (*n* = 17).
**TABLE S6.** Syndromes at the time of presurgical evaluation. Generalized estimating equation odds ratios (ORs) with 95% confidence intervals are shown for infantile epileptic spasms syndrome (IESS), developmental and epileptic encephalopathy with spike‐and‐wave activation in sleep (DEE‐SWAS), and Lennox–Gastaut syndrome (LGS) at presurgical evaluation, comparing each continent with Europe. OR < 1 indicates a lower prevalence and OR > 1 a higher prevalence than Europe. The intercept reflects Europe's baseline odds. Statistically significant findings (*p* < .05) are shown in bold. Africa was excluded due to the small sample size (*n* = 17).
**TABLE S7.** Diagnostic modalities used per patient by continent. Generalized estimating equation estimates compare the number of diagnostic modalities used per patient across continents, with Europe as the reference. Entries represent estimated differences with 95% confidence intervals; negative values indicate fewer modalities and positive values more modalities than Europe. The intercept reflects Europe's baseline estimate. Statistically significant results (*p* < .05) are shown in bold. Africa was excluded due to the small sample size (*n* = 17).
**TABLE S8.** Use of specific presurgical investigations by continent. Generalized estimating equation odds ratios (ORs) with 95% confidence intervals for individual modalities—functional magnetic resonance imaging (fMRI; language/motor), interictal fluorodeoxyglucose positron emission tomography (FDG‐PET), other PET, ictal single photon emission computed tomography (SPECT), magnetoencephalography (MEG), Wada test, genetic testing, electroencephalography (EEG)–fMRI, MRI postprocessing, EEG source localization, and high‐density (hd)‐EEG—compare each continent with Europe. OR < 1 indicates lower use and OR > 1 higher use than Europe. The intercept reflects Europe's baseline odds. Statistically significant values (*p* < .05) are shown in bold. Africa was excluded from the analysis due to the small sample size (*n* = 17).

## Data Availability

The data that support the findings of this study are available from the corresponding author upon reasonable request.

## APPENDIX A

### A.1 ILAE Pediatric Epilepsy Surgery Task Force and Study Group

Fabrice Bartolomei, Juan Carlos Benedetti Isaac, Poodipedi Sarat Chandra, Mary Connoly, Daniel Curry, Vicki Deli‐Peri, Luca De Palma, Nabeel Diab, Petia Dimova, Carolina Munoz Castro, Georg Dorfmüller, Jana Domínguez, Gianluca D'Onofrio, Christin Eltze, Johannes M. Nico Enslin, András Fogarasi, Siby Gopinath, Renzo Guerrini, Giulia Nobile, Masaki Iwasaki, Sita Jayalakshmi, Takayuki Kikuchi, Noritsugu Kunihiro, Paweł Kowalczyk, Niklaus Krayenbühl, Pavel Kršek, Thilo Kalbhenn, Kensuke Kawai, Cassia Maniquis, Wim Otte, María Ángeles Pérez‐Jiménez, Juan Carlos Pérez‐Poveda, Catherine Riney, Jonathan Roth, Sabine Rona, Karl Rössler, Olga Teo, Oana Tarta Arsene, Laura Tassi, Hartlieb Till, Dhanya T S, Catalina Soto Órdenes, Piradee Suwanpakdee, Steffen Syrbe, Nigel Peter Symss, Jay Shetty, Dilşad Türkdoğan, Takehiro Uda, Naotaka Usui, Vrajesh Udani, Jithangi Wanigasinghe, Yi Wang.

### A.2 Study group author emails and ORCID numbers

- Fabrice Bartolomei; fabrice.bartolomei@ap-hm.fr; 0000–0002–1678‐0297
- Juan Carlos Benedetti Isaac; benedetti.jc@gmail.com; 0000–0002–2307‐6724
- Poodipedi Sarat Chandra; saratpchandra3@gmail.com; 0000–0002–3375‐6803
- Mary Connoly; mconnolly@cw.bc.ca; 0000–0002‐7784‐942X
- Daniel Curry; djcurry@bcm.edu; 0000–0003–3661‐2976
- Gianluca D'Onofrio; gianluca.donofrio@institutimagine.org; 0000–0003–4987‐5919
- Luca De Palma; luca.depalma@opbg.net; 0000–0002–0714‐8230
- Vicki Deli‐Peri; vickideliperi@icloud.com; 0000–0003‐2640‐249X
- Nabeel Diab; nabeel.diab@bcm.edu
- Petia Dimova; psdimova@gmail.com; 0000–0002–4443‐5140
- Jana Domínguez; jana.dominguez@sjd.es; 0000–0002–7197‐7391
- Georg Dorfmüller; gdorfmuller@for.paris; 0000–0002–4503‐6184
- Christin Eltze; christin.eltze@gosh.nhs.uk; 0000–0003–4411‐2939
- Johannes M. Nico Enslin; johannes.enslin@uct.ac.za; 0000–0002–6384‐7781
- András Fogarasi; fog.andras@gmail.com
- Siby Gopinath; sibygopi@gmail.com; 0000–0002–3333‐0933
- Renzo Guerrini; renzo.guerrini@meyer.it; 0000–0002–7272‐7079
- Masaki Iwasaki; iwa@ncnp.go.jp; 0000–0002–4204‐7144
- Sita Jayalakshmi; sita_js@hotmail.com; 0000–0001–8630‐0896
- Thilo Kalbhenn; thilo.kalbhenn@evkb.de; 0000–0001‐6249‐629X
- Kensuke Kawai; kenkawai_tky@me.com; 0000–0002–8218‐7360
- Takayuki Kikuchi; tkik@kuhp.kyoto-u.ac.jp; 0000–0002–6295‐5510
- Paweł Kowalczyk; p.kowalczyk@ipczd.pl
- Niklaus Krayenbühl; niklaus.krayenbuehl@kispi.uzh.ch; 0000–0003‐1089‐221X
- Pavel Kršek; pavel.krsek@post.cz; 0000–0002–5740‐3690
- Noritsugu Kunihiro; nori9216@omu.ac.jp; 0009–0002–1294‐2938
- Cassia Maniquis; cmaniquis@mednet.ucla.edu
- Carolina Munoz Castro; caromunc@gmail.com; 0009–0003–4208‐2892
- Giulia Nobile; giulianobile@gaslini.org; 0000–0002–9957‐7760
- Wim Otte; wmotte@gmail.com; 0000–0003–1511‐6834
- María Ángeles Pérez‐Jiménez; mpjimenez@salud.madrid.org; 0000–0003–0311‐0531
- Juan Carlos Pérez‐Poveda; jcperez@husi.org.co / j-perez@javeriana.edu.co; 0000–0001–5360‐5650
- Catherine Riney; kate.riney@health.qld.gov.au; 0000–0002–1122‐3555
- Sabine Rona; sabine.rona@t-online.de
- Karl Rössler; karl.roessler@meduniwien.ac.at; 0000–0002–9249‐8082
- Jonathan Roth; jonaroth@gmail.com; 0000–0002–4267‐8835
- Jay Shetty; jay.shetty@ed.ac.uk; 0000–0002–3114‐4002
- Catalina Soto Órdenes; dra.karinarosso@gmail.com
- Piradee Suwanpakdee; piradee@pedpmk.org; 0000–0001–9814‐3867
- Nigel Peter Symss; nigelpetersymss@gmail.com; 0009–0006‐7431‐416X
- Steffen Syrbe; steffen.syrbe@med.uni-heidelberg.de; 0000–0003–2543‐4844
- Dhanya T S; dhanyats16@gmail.com
- Oana Tarta Arsene; otartaarsene@yahoo.com; 0000–0002‐9690‐456X
- Laura Tassi; laura.tassi@ospedaleniguarda.it; 0000–0002–0632‐7296
- Olga Teo; olga.teo@health.nsw.gov.au; 0009–0001–3123‐9159
- Hartlieb Till; thartlieb@schoen-kliniken.de
- Dilşad Türkdoğan; dturkdogan@hotmail.com; 0000–0002–6607‐5860
- Takehiro Uda; udat@omu.ac.jp; 0000–0001–6422‐4140
- Vrajesh Udani; vrajesh.udani@gmail.com; 0000–0002–8523‐8215
- Naotaka Usui; usuin_0209@yahoo.co.jp; 0000–0002–9736‐7460
- Yi Wang; wangyi19931027@sina.com; 0000–0002–4775‐5457
- Jithangi Wanigasinghe; jithangi@gmail.com; 0000–0002–9413‐8363

## References

[1] Dwivedi R , Ramanujam B , Chandra PS , Sapra S , Gulati S , Kalaivani M , et al. Surgery for drug‐resistant epilepsy in children. N Engl J Med. 2017;377(17):1639–1647. 29069568 10.1056/NEJMoa1615335

[2] Widjaja E , Jain P , Demoe L , Guttmann A , Tomlinson G , Sander B . Seizure outcome of pediatric epilepsy surgery: systematic review and meta‐analyses. Neurology. 2020;94(7):311–321. 31996452 10.1212/WNL.0000000000008966

[3] Barba C , Cross JH , Braun K , Cossu M , Klotz KA , De Masi S , et al. Trends in pediatric epilepsy surgery in Europe between 2008 and 2015: country‐, center‐, and age‐specific variation. Epilepsia. 2020;61(2):216–227. 31876960 10.1111/epi.16414

[4] Vessell M , Willett A , Ugiliweneza B , Sharma M , Mutchnick I , Boakye M , et al. National 22‐year epilepsy surgery landscape shows increasing open and minimally invasive pediatric epilepsy surgery. Epilepsia. 2024;65(8):2423–2437. 38943543 10.1111/epi.18030

[5] Barba C , Specchio N , Guerrini R , Tassi L , De Masi S , Cardinale F , et al. Increasing volume and complexity of pediatric epilepsy surgery with stable seizure outcome between 2008 and 2014: a nationwide multicenter study. Epilepsy Behav. 2017;75:151–157. 28866334 10.1016/j.yebeh.2017.08.010

[6] Asranna A , Babu Pasangulapati S , Menon R , Radhakrishnan A . Trends in pediatric epilepsy surgery: a lower‐middle‐income country perspective. Acta Neurol Scand. 2021;143(5):521–529. 33438764 10.1111/ane.13393

[7] Baud MO , Perneger T , Rácz A , Pensel MC , Elger C , Rydenhag B , et al. European trends in epilepsy surgery. Neurology. 2018;91(2):e96–e106. 29898967 10.1212/WNL.0000000000005776

[8] Lamberink HJ , Boshuisen K , van Rijen PC , Gosselaar PH , Braun KPJ , Dutch Collaborative Epilepsy Surgery Program (DCESP) . Changing profiles of pediatric epilepsy surgery candidates over time: a nationwide single‐center experience from 1990 to 2011. Epilepsia. 2015;56(5):717–725. 25847357 10.1111/epi.12974

[9] Eriksson MH , Whitaker KJ , Booth J , Piper RJ , Chari A , Martin Sanfilippo P , et al. Pediatric epilepsy surgery from 2000 to 2018: changes in referral and surgical volumes, patient characteristics, genetic testing, and postsurgical outcomes. Epilepsia. 2023;64(9):2260–2273. 37264783 10.1111/epi.17670 PMC7615891

[10] Harvey AS , Cross JH , Shinnar S , Mathern GW , ILAE Pediatric Epilepsy Surgery Survey Taskforce . Defining the spectrum of international practice in pediatric epilepsy surgery patients. Epilepsia. 2008;49(1):146–155. 18042232 10.1111/j.1528-1167.2007.01421.x

[11] Ostendorf AP , Ahrens SM , Lado FA , Arnold ST , Bai S , Bensalem Owen MK , et al. United States epilepsy center characteristics: a data analysis from the National Association of epilepsy centers. Neurology. 2022;98(5):e449–e458. 34880093 10.1212/WNL.0000000000013130 PMC8826463

[12] Kadish NE , Bast T , Reuner G , Wagner K , Mayer H , Schubert‐Bast S , et al. Epilepsy surgery in the first 3 years of life: predictors of seizure freedom and cognitive development. Neurosurgery. 2019;84(6):E368–E377. 30137548 10.1093/neuros/nyy376

[13] Makridis KL , Klotz KA , Ramantani G , Becker L‐L , San Antonio‐Arce V , Syrbe S , et al. Epilepsy surgery in early infancy: a retrospective, multicenter study. Epilepsia Open. 2023;8(3):1182–1189. 37458529 10.1002/epi4.12791 PMC10472416

[14] Roth J , Constantini S , Ekstein M , Weiner HL , Tripathi M , Chandra PS , et al. Epilepsy surgery in infants up to 3 months of age: safety, feasibility, and outcomes: a multicenter, multinational study. Epilepsia. 2021;62(8):1897–1906. 34128544 10.1111/epi.16959

[15] Ramantani G , Wirrell E . Epilepsy surgery in developmental and epileptic encephalopathies. Epilepsy Behav. 2024;159:109985. 39181112 10.1016/j.yebeh.2024.109985

[16] Sanders MWCB , Lemmens CMC , Jansen FE , Brilstra EH , Koeleman BPC , Braun KPJ , et al. Implications of genetic diagnostics in epilepsy surgery candidates: a single‐center cohort study. Epilepsia Open. 2019;4(4):609–617. 31819917 10.1002/epi4.12366 PMC6885658

[17] Alsubhi S , Berrahmoune S , Dudley RWR , Dufresne D , Simard Tremblay E , Srour M , et al. Utility of genetic testing in the pre‐surgical evaluation of children with drug‐resistant epilepsy. J Neurol. 2024;271(5):2503–2508. 38261030 10.1007/s00415-023-12174-3

[18] Bernasconi A , Cendes F , Theodore WH , Gill RS , Koepp MJ , Hogan RE , et al. Recommendations for the use of structural magnetic resonance imaging in the care of patients with epilepsy: a consensus report from the international league against epilepsy neuroimaging task force. Epilepsia. 2019;60(6):1054–1068. 31135062 10.1111/epi.15612

[19] D'Gama AM , Poduri A . Brain somatic mosaicism in epilepsy: bringing results back to the clinic. Neurobiol Dis. 2023;181:106104. 36972791 10.1016/j.nbd.2023.106104

[20] Jehi L , Jette N , Kwon C‐S , Josephson CB , Burneo JG , Cendes F , et al. Timing of referral to evaluate for epilepsy surgery: expert consensus recommendations from the surgical therapies Commission of the International League against Epilepsy. Epilepsia. 2022;63(10):2491–2506. 35842919 10.1111/epi.17350 PMC9562030

[21] Mouthaan BE , Rados M , Barsi P , Boon P , Carmichael DW , Carrette E , et al. Current use of imaging and electromagnetic source localization procedures in epilepsy surgery centers across Europe. Epilepsia. 2016;57(5):770–776. 27012361 10.1111/epi.13347

[22] Gaillard WD , Jette N , Arnold ST , Arzimanoglou A , Braun KPJ , Cukiert A , et al. Establishing criteria for pediatric epilepsy surgery center levels of care: report from the ILAE pediatric epilepsy surgery task force. Epilepsia. 2020;61(12):2629–2642. 33190227 10.1111/epi.16698

[23] R Core Team . R: The R Project for Statistical Computing. 2022 https://www.r‐project.org/

[24] World Bank Data Help Desk . World Bank Country and Lending Groups. 2026 https://datahelpdesk.worldbank.org/knowledgebase/articles/906519

[25] Cross JH , Reilly C , Gutierrez Delicado E , Smith ML , Malmgren K . Epilepsy surgery for children and adolescents: evidence‐based but underused. Lancet Child Adolesc Health. 2022;6(7):484–494. 35568054 10.1016/S2352-4642(22)00098-0

[26] Cross JH , Jayakar P , Nordli D , Delalande O , Duchowny M , Wieser HG , et al. Proposed criteria for referral and evaluation of children for epilepsy surgery: recommendations of the subcommission for pediatric epilepsy surgery. Epilepsia. 2006;47(6):952–959. 16822241 10.1111/j.1528-1167.2006.00569.x

[27] Hale AT , Chari A , Scott RC , Helen Cross J , Rozzelle CJ , Blount JP , et al. Expedited epilepsy surgery prior to drug resistance in children: a frontier worth crossing? Brain. 2022;145(11):3755–3762. 35883201 10.1093/brain/awac275

[28] Ramantani G . Epilepsy surgery in early life: the earlier, the better. World Neurosurg. 2019;131:285–286. 31658555 10.1016/j.wneu.2019.08.084

[29] Pestana Knight EM , Schiltz NK , Bakaki PM , Koroukian SM , Lhatoo SD , Kaiboriboon K . Increasing utilization of pediatric epilepsy surgery in the United States between 1997 and 2009. Epilepsia. 2015;56(3):375–381. 25630252 10.1111/epi.12912 PMC4363272

[30] Hatoum R , Nathoo‐Khedri N , Shlobin NA , Wang A , Weil AG , Fallah A . Barriers to epilepsy surgery in pediatric patients: a scoping review. Seizure. 2022;102:83–95. 36209677 10.1016/j.seizure.2022.08.013

[31] Bui K‐TA , Wahby S , Jetté N , Bouthillier A , Hader WJ , Keezer MR , et al. Inequalities in the utilisation of epilepsy surgery for adults and children in Canada. Epilepsy Res. 2018;148:63–68. 30390542 10.1016/j.eplepsyres.2018.10.006

[32] Englot DJ . Lesional epilepsy in children: removing doubt to cut it out. Epilepsy Curr. 2023;23(3):150–152. 37334412 10.1177/15357597231156735 PMC10273808

[33] Berl MM , Wagner J , Caraway A , Loblein H , Novotny EJ , Patrick K , et al. Disparities across the pediatric epilepsy surgery journey: referral, recommendation, and completion from a national consortium. Epilepsia. 2026;67:1601–1613. 41579031 10.1002/epi.70060

[34] Ramantani G , Kadish NE , Strobl K , Brandt A , Stathi A , Mayer H , et al. Seizure and cognitive outcomes of epilepsy surgery in infancy and early childhood. Eur J Paediatr Neurol. 2013;17(5):498–506. 23602440 10.1016/j.ejpn.2013.03.009

[35] Ramantani G , Stathi A , Brandt A , Strobl K , Schubert‐Bast S , Wiegand G , et al. Posterior cortex epilepsy surgery in childhood and adolescence: predictors of long‐term seizure outcome. Epilepsia. 2017;58(3):412–419. 28098941 10.1111/epi.13654

[36] Ramantani G , Kadish NE , Anastasopoulos C , Brandt A , Wagner K , Strobl K , et al. Epilepsy surgery for glioneuronal tumors in childhood: avoid loss of time. Neurosurgery. 2014;74(6):648–657. 24584135 10.1227/NEU.0000000000000327

[37] Maillard LG , Tassi L , Bartolomei F , Catenoix H , Dubeau F , Szurhaj W , et al. Stereoelectroencephalography and surgical outcome in polymicrogyria‐related epilepsy: a multicentric study. Ann Neurol. 2017;82(5):781–794. 29059488 10.1002/ana.25081

[38] Ramantani G , Kadish NE , Mayer H , Anastasopoulos C , Wagner K , Reuner G , et al. Frontal lobe epilepsy surgery in childhood and adolescence: predictors of long‐term seizure freedom, overall cognitive and adaptive functioning. Neurosurgery. 2018;83(1):93–103. 29106684 10.1093/neuros/nyx340

[39] Perucca E , Perucca P , White HS , Wirrell EC . Drug resistance in epilepsy. Lancet Neurol. 2023;22(8):723–734. 37352888 10.1016/S1474-4422(23)00151-5

[40] Yossofzai O , Biswas A , Moineddin R , Ibrahim GM , Rutka J , Donner E , et al. Number of epilepsy surgeries has decreased despite an increase in pre‐surgical evaluations at a tertiary pediatric epilepsy center in Ontario. Seizure‐Eur J Epilep. 2023;108:1–9. 10.1016/j.seizure.2023.04.001 37059033

[41] Braun KPJ , Cross JH . Pediatric epilepsy surgery: the earlier the better. Expert Rev Neurother. 2018;18(4):261–263. 29560752 10.1080/14737175.2018.1455503

[42] Giulioni M , Marucci G , Pelliccia V , Gozzo F , Barba C , Didato G , et al. Epilepsy surgery of “low grade epilepsy associated neuroepithelial tumors”: a retrospective nationwide Italian study. Epilepsia. 2017;58(11):1832–1841. 28804898 10.1111/epi.13866

[43] Pelliccia V , Deleo F , Gozzo F , Giovannelli G , Mai R , Cossu M , et al. Early epilepsy surgery for non drug‐resistant patients. Epilepsy Behav Rep. 2022;19:100542. 35573058 10.1016/j.ebr.2022.100542 PMC9096667

[44] Cohen NT , Chang P , You X , Zhang A , Havens KA , Oluigbo CO , et al. Prevalence and risk factors for Pharmacoresistance in children with focal cortical dysplasia‐related epilepsy. Neurology. 2022;99(18):e2006–e2013. 35985831 10.1212/WNL.0000000000201033 PMC9651467

[45] Lamberink HJ , Otte WM , Blümcke I , Braun KPJ , Aichholzer M , Amorim I , et al. Seizure outcome and use of antiepileptic drugs after epilepsy surgery according to histopathological diagnosis: a retrospective multicentre cohort study. Lancet Neurol. 2020;19(9):748–757. 32822635 10.1016/S1474-4422(20)30220-9

[46] Bjellvi J , Olsson I , Malmgren K , Wilbe Ramsay K . Epilepsy duration and seizure outcome in epilepsy surgery: a systematic review and meta‐analysis. Neurology. 2019;93(2):e159–e166. 31182508 10.1212/WNL.0000000000007753 PMC6656653

[47] Lewis AK , Taylor NF , Carney PW , Harding KE . What is the effect of delays in access to specialist epilepsy care on patient outcomes? A systematic review and meta‐analysis. Epilepsy Behav. 2021;122:108192. 34265620 10.1016/j.yebeh.2021.108192

[48] Stefanos‐Yakoub I , Wingeier K , Held U , Latal B , Wirrell E , Smith ML , et al. Long‐term intellectual and developmental outcomes after pediatric epilepsy surgery: a systematic review and meta‐analysis. Epilepsia. 2024;65(2):251–265. 38031640 10.1111/epi.17834

[49] Schmidlechner T , Zaddach M , Heinen F , Cornell S , Ramantani G , Rémi J , et al. IQ changes after pediatric epilepsy surgery: a systematic review and meta‐analysis. J Neurol. 2024;271(1):177–187. 37770569 10.1007/s00415-023-12002-8 PMC10770207

[50] Eriksson MH , Prentice F , Piper RJ , Wagstyl K , Adler S , Chari A , et al. Long‐term neuropsychological trajectories in children with epilepsy: does surgery halt decline? Brain. 2024;147(8):2791–2802. 38643018 10.1093/brain/awae121 PMC11292899

[51] Boshuisen K , van Schooneveld MMJ , Uiterwaal CSPM , Cross JH , Harrison S , Polster T , et al. Intelligence quotient improves after antiepileptic drug withdrawal following pediatric epilepsy surgery. Ann Neurol. 2015;78(1):104–114. 25899932 10.1002/ana.24427

[52] Cloppenborg T , van Schooneveld M , Hagemann A , Hopf JL , Kalbhenn T , Otte WM , et al. Development and validation of prediction models for developmental and intellectual outcome following pediatric epilepsy surgery. Neurology. 2022;98(3):e225–e235. 34795046 10.1212/WNL.0000000000013065

[53] Ramantani G , Strobl K , Stathi A , Brandt A , Schubert‐Bast S , Wiegand G , et al. Reoperation for refractory epilepsy in childhood: a second chance for selected patients. Neurosurgery. 2013;73(4):695–704. 23842559 10.1227/NEU.0000000000000081

[54] Bicciato G , Gennari AG , Oertel MF , Dünner C , Krayenbühl N , Boltshauser E , et al. Laser interstitial thermal therapy in pediatric cerebellar epilepsy. Epileptic Disord. 2023;25(6):880–885. 37584626 10.1002/epd2.20149

[55] Blumcke I , Budday S , Poduri A , Lal D , Kobow K , Baulac S . Neocortical development and epilepsy: insights from focal cortical dysplasia and brain tumours. Lancet Neurol. 2021;20(11):943–955. 34687638 10.1016/S1474-4422(21)00265-9

[56] Barba C , Mai R , Grisotto L , Gozzo F , Pellacani S , Tassi L , et al. Unilobar surgery for symptomatic epileptic spasms. Ann Clin Transl Neurol. 2017;4(1):36–45. 28078313 10.1002/acn3.373 PMC5221449

[57] Ramantani G , Bölsterli BK , Alber M , Klepper J , Korinthenberg R , Kurlemann G , et al. Treatment of infantile spasm syndrome: update from the interdisciplinary guideline committee coordinated by the German‐speaking Society of Neuropediatrics. Neuropediatrics. 2022;53(6):389–401. 35882373 10.1055/a-1909-2977 PMC9643068

[58] Boßelmann CM , San Antonio‐Arce V , Schulze‐Bonhage A , Fauser S , Zacher P , Mayer T , et al. Genetic testing before epilepsy surgery ‐ an exploratory survey and case collection from German epilepsy centers. Seizure. 2022;95:4–10. 34953286 10.1016/j.seizure.2021.12.004

[59] Cai L , Zhang K , Zhou W , Shao X , Guan Y , Yu T , et al. Consensus on pediatric epilepsy surgery for young children: an investigation by the China association against epilepsy task force on epilepsy surgery. Acta Epileptol. 2023;5(1):20. 40217282 10.1186/s42494-023-00130-7 PMC11960312

[60] Jayakar P , Gaillard WD , Tripathi M , Libenson MH , Mathern GW , Cross JH , et al. Diagnostic test utilization in evaluation for resective epilepsy surgery in children. Epilepsia. 2014;55(4):507–518. 24512473 10.1111/epi.12544

[61] Garcia‐Uzquiano R , Barcia G , Losito E , Chemaly N , Kaminska A , Desguerre I , et al. Genetic testing, another important tool in presurgical evaluation of focal epilepsies in childhood. Epilepsia Open. 2024;9(4):1589–1596. 38829689 10.1002/epi4.12964 PMC11296104

[62] Checri R , Chipaux M , Ferrand‐Sorbets S , Raffo E , Bulteau C , Rosenberg SD , et al. Detection of brain somatic mutations in focal cortical dysplasia during epilepsy presurgical workup. Brain Commun. 2023;5(3):fcad174. 37324239 10.1093/braincomms/fcad174 PMC10261848

[63] Gerasimenko A , Baldassari S , Baulac S . mTOR pathway: insights into an established pathway for brain mosaicism in epilepsy. Neurobiol Dis. 2023;182:106144. 37149062 10.1016/j.nbd.2023.106144

[64] López‐Rivera JA , Leu C , Macnee M , Khoury J , Hoffmann L , Coras R , et al. The genomic landscape across 474 surgically accessible epileptogenic human brain lesions. Brain. 2023;146(4):1342–1356. 36226386 10.1093/brain/awac376 PMC10115236

[65] Sanders MWCB , Koeleman BPC , Brilstra EH , Jansen FE , Baldassari S , Chipaux M , et al. Somatic variant analysis of resected brain tissue in epilepsy surgery patients. Epilepsia. 2024;65(12):e209–e215. 39460693 10.1111/epi.18148 PMC11647421

[66] Schuele S , Busch RM , Frauscher B , Gnatkovsky V , Hamer H , Jehi L , et al. Seminars in epileptology: Presurgical epilepsy evaluation. Epileptic Disord. 2025;27(6):1105–1147. 40991343 10.1002/epd2.70105 PMC12747706

[67] Gennari AG , Waelti S , Schwyzer M , Treyer V , Rossi A , Sartoretti T , et al. Long‐term trends in total administered radiation dose from brain [18F]FDG‐PET in children with drug‐resistant epilepsy. Eur J Nucl Med Mol Imaging. 2025;52(2):574–585. 39352423 10.1007/s00259-024-06902-8 PMC11732939

[68] Santalucia R , Carosio C , Gennari AG , Baroumand AG , Vrielynck P , Fierain A , et al. Combining interictal and ictal low‐density EEG source imaging to delineate the epileptogenic zone in young children. Neurotherapeutics. 2026;23(1):e00821. 41419421 10.1016/j.neurot.2025.e00821 PMC12976537

[69] Bast T , Ramantani G , Boppel T , Metzke T , Ozkan O , Stippich C , et al. Source analysis of interictal spikes in polymicrogyria: loss of relevant cortical fissures requires simultaneous EEG to avoid MEG misinterpretation. NeuroImage. 2005;25(4):1232–1241. 15850741 10.1016/j.neuroimage.2004.12.059

[70] Jäger V , Dümpelmann M , LeVan P , Ramantani G , Mader I , Schulze‐Bonhage A , et al. Concordance of epileptic networks associated with epileptic spikes measured by high‐density EEG and fast fMRI. PLoS One. 2015;10(10):e0140537. 26496480 10.1371/journal.pone.0140537 PMC4619722

[71] Jacobs J , Stich J , Zahneisen B , Assländer J , Ramantani G , Schulze‐Bonhage A , et al. Fast fMRI provides high statistical power in the analysis of epileptic networks. NeuroImage. 2014;88:282–294. 24140936 10.1016/j.neuroimage.2013.10.018

