# Supplementary material for: Pediatric epilepsy surgery: Global survey of referral and presurgical evaluation practices
Source: Epilepsia. 2026 Jun 2;67(8):4008–24. doi: 10.1002/epi.70312 (PMC13525590; doi:10.1002/epi.70312)
Supplement: Supplementary file 1 [file EPI-67-4008-s001.docx]

**SUPPLEMENTARY MATERIAL**

**SUPPLEMENTARY FIGURE**

**Supplementary Figure 1. History of epilepsy syndromes by continent.**

Bar plots show the proportion of patients with a documented history of infantile epileptic spasms syndrome (IESS), Lennox–Gastaut syndrome (LGS), developmental epileptic encephalopathy with spike-and-wave activation in sleep (DEE-SWAS), or other defined epilepsy syndromes. Globally, 24.8% of patients had a history of an epilepsy syndrome. IESS was the most frequently reported historical syndrome (12.3%), followed by LGS (6.2%) and DEE-SWAS (1.6%). Compared with Europe, a history of LGS was more common in South America, while DEE-SWAS was less frequent in Oceania, North America, and South America. No significant regional differences were observed for IESS. Bar width is proportional to the number of patients per region. Gray background bars indicate global usage rates.

**SUPPLEMENTARY TABLES**

| Institution | Responsible Physicians | Location |
| --- | --- | --- |
| ***Africa (n=17)*** |  |  |
| Mediclinic Constantiaberg | James Butler, Vicki Deli-Peri | Cape Town, South Africa |
| Red Cross War Memorial Children's Hospital | Jo Wilmshurst, Johannes M. Nico Enslin, Richard J. Burman | Cape Town, South Africa |
| ***Asia (n=960)*** |  |  |
| Epilepsy Center, Shenzhen Children's Hospital | Dezhi Cao | Shenzhen, China |
| Pediatric Epilepsy Center, Peking University First Hospital | Lixin Cai, Yuwu Jiang | Beijing, China |
| Krishna Institute of Medical Sciences | Sita Jayalakshmi, Manas Panigrahi | Secunderabad, India |
| Epilepsy Center, National Center of Neurology and Psychiatry | Masaki Iwasaki | Tokyo, Japan |
| All India Institute of Medical Sciences (AIIMS) | Manjari Tripathi, Poodipedi Sarat Chandra | Delhi, India |
| Deenanath Mangeshkar Hospital | Sandeep Bhagwan Patil | Pune, India |
| National Epilepsy Center, NHO Shizuoka Institute of Epilepsy and Neurological Disorders | Naotaka Usui | Shizuoka, Japan |
| Amrita Advanced Centre for Epilepsy (AACE), Amrita Institute of Medical Sciences, Amrita Vishwa Vidyapeetham | Siby Gopinath, Ashok Pillai | Kochi, Kerala, India |
| Osaka City General Hospital | Noritsugu Kunihiro, Takehiro Uda | Osaka, Japan |
| Gleneagles Hospital | Kannan Lakshminarayanan, Nigel Peter Symss | Chennai, Tamilnadu, India |
| P.D. Hinduja National Hospital & Medical Research Centre | Vrajesh Udani | Mumbai, India |
| Phramongkutklao Hospital | Piradee Suwanpakdee, Siraruj Sakoolnamarka | Bangkok, Thailand |
| Kyoto University Graduate School of Medicine | Takayuki Kikuchi | Kyoto, Japan |
| Osaka Metropolitan University Hospital | Takehiro Uda | Osaka, Japan |
| Avitis Institute of Medical Sciences | Dhanya T S, Kurupath Radhakrishnan | Palakkad, Kerala, India |
| Jichi Medical University Hospital | Kensuke Kawai, Naoto Kunii | Shimotsuke, Tochigi, Japan |
| National Epilepsy Center, National Hospital of Sri Lanka | Jithangi Wanigasinghe, Sanjaya Fernando, Sanjeewa Garusinghe | Colombo, Sri Lanka |
| ***Oceania (n=79)*** |  |  |
| The Royal Children's Hospital | Simon Harvey | Melbourne, Australia |
| The Children's Hospital at Westmead | Deepak Gill, Sachin Gupta | Sydney, Australia |
| Queensland Children's Hospital | Sameer Dal, Steve Malone, Kate Riney | Brisbane, Australia |
| ***Europe (n=795)*** |  |  |
| Great Ormond Street Hospital for Children | Helen Cross, Christin Eltze, Suresh Pujar, Martin Tisdall, Zubair Tahir, Friederike Moeller | London, UK |
| Royal Hospital for Children and Young People | Jay Shetty, Ailsa McLellan | Edinburgh, UK |
| Rothschild Foundation Hospital | Sarah Ferrand Sorbets, Georg Dorfmüller, Mathilde Chipaux Raffo | Paris, France |
| Bethel Epilepsy Center | Thomas Cloppenborg, Thilo Kalbhenn | Bielefeld, Germany |
| Hospital Sant Joan de Déu | Alexis Arzimanoglou, Javier Aparicio, Jana Domínguez, Alia Ramírez, Carlos Valera | Barcelona, Spain |
| The Children's Memorial Health Institute | Katarzyna Kotulska-Jóźwiak, Paweł Kowalczyk | Warsaw, Poland |
| University Medical Center Utrecht | Kees Braun | Utrecht, Netherlands |
| Medical University of Vienna | Martha Feucht, Karl Rössler | Vienna, Austria |
| Claudio Munari' Epilepsy Surgery Centre, ASST Niguarda Hospital | Laura Tassi, Veronica Pelliccia | Milan, Italy |
| Charles University and Motol and Homolka University Hospital and EpiReC Consortium | Martin Kudr, Pavel Krsek | Prague, Czech Republic |
| Bambino Gesù Children's Hospital | Nicola Specchio, Luca de Palma | Rome, Italy |
| Niño Jesús University Hospital | María Ángeles Pérez Jiménez, Marcelo Budke | Madrid, Spain |
| Tel Aviv Medical Center | Jonathan Roth, Shimrit Uliel-Sibony | Tel Aviv, Israel |
| Freiburg Epilepsy Center | Victoria San Antonio-Arce, Andreas Schulze-Bonhage | Freiburg, Germany |
| Center for Pediatric Neurology, Neurorehabilitation, and Epileptology, Schoen-Clinic | Tom Pieper, Till Hartlieb, Manfred Kudernatsch | Vogtareuth, Germany |
| Meyer Children's Hospital IRCCS, and University of Florence | Carmen Barba, Renzo Guerrini, Flavio Giordano | Florence, Italy |
| Clinical Hospital 'Alexandru Obregia' | Oana Tarta Arsene | Bucharest, Romania |
| Istituto Giannina Gaslini Children’s Hospital, IRCCS, University of Genoa | Lino Nobili, Stefano Francione, Giulia Nobile, Alessandro Consales | Genoa, Italy |
| University Children's Hospital Zurich | Georgia Ramantani, Niklaus Krayenbühl | Zürich, Switzerland |
| Queen Silvia Children’s Hospital, Sahlgrenska University Hospital | Tove Hallböök | Gothenburg, Sweden |
| La Timone Hospital, Aix-Marseille University | Fabrice Bartolomei, Didier Scavarda | Marseille, France |
| Department of Pediatric Neurology and Epilepsy Surgery Center, Marmara University | Dilşad Türkdoğan, Adnan Dağçinar | İstanbul, Turkey |
| University Hospital St. Ivan Rilski | Petia Dimova | Sofia, Bulgaria |
| Bethesda Children's Hospital | Lejla Vendegh, András Fogarasi | Budapest, Hungary |
| ***North America (n=427)*** |  |  |
| Texas Children’s Hospital | Howard Weiner, Kalman Katlowitz, Daniel Curry | Houston, TX, USA |
| The Hospital for Sick Children | Mary Lou Smith | Toronto, Canada |
| Nicklaus Children's Hospital | Matt Lallas | Miami, FL, USA |
| Epilepsy Center, Cleveland Clinic | Ahsan Moosa Naduvil Valappil, William Bingaman | Cleveland, OH, USA |
| Mayo Clinic | Elaine Wirrell | Rochester, MN, USA |
| David Geffen School of Medicine at UCLA | Aria Fallah, Cassia Maniquis | Los Angeles, CA, USA |
| British Columbia´s Children's Hospital | Mary Connolly, Mandeep Tamber | Vancouver, BA, Canada |
| CHU Sainte Justine, Université de Montréal | Aris Hadjinicolaou, Gianluca D'Onofrio, Alexander Weil | Montreal, Canada |
| ***South America (n=149)*** |  |  |
| Clinica Cukiert | Arthur Cukiert, Cristine Cukiert | Sao Paulo, Brazil |
| Hospital de Pediatría "Prof. Dr. Juan P Garrahan" | Roberto Caraballo | Buenos Aires, Argentina |
| Guillermo Grant Benavente Hospital | Carolina Munoz Castro | Concepcion, Chile |
| Hospital Carlos Van Buren, University of Valparaíso | Catalina Soto Órdenes | Valparaíso, Chile |
| Hospital Universitario San Ignacio | Juan Carlos Perez Poveda, Oscar Zorro Guio, Daniel Nariño González | Bogotá, Colombia |
| Fundación Hospital Pediátrico la Misericordia | Laura Guio Mahecha, Oscar Zorro Guio, Juan Carlos Perez Poveda, Daniel Nariño González | Bogotá, Colombia |
| Clínica Portoazul Auna | Juan Carlos Perez Poveda, Benedetti Isaac | Barranquilla, Colombia |

**Supplementary Table 1.** Pediatric epilepsy surgery centers and investigators participating in the 2023 ILAE survey.

| Continent | Age at epilepsy onset | Age at surgery | Epilepsy duration | Surgery before pharmacoresistance | Surgery in the first year of life |
| --- | --- | --- | --- | --- | --- |
| (Intercept) | 3.97 (3.53-4.41), p<0.001 | 9.43 (8.71-10.15), p<0.001 | 5.46 (4.56-6.35), p<0.001 | 0.18 (0.08–0.38), p<0.001 | 0.03 (0.02–0.05), p<0.001 |
| Asia | -0.64 (-1.37-0.09), p=0.084 | **-1.48 (-2.87--0.08), p=0.039** | -0.83 (-2.4-0.74), p=0.298 | not estimable | 1.12 (0.62–2.01), p=0.710 |
| North America | 0.01 (-0.76-0.79), p=0.975 | **1.14 (0.11-2.18), p=0.031** | **1.13 (0.13-2.13), p=0.027** | **0.12 (0.05–0.28), p<0.001** | 0.56 (0.15–2.11), p=0.396 |
| Oceania | 0.26 (-1.17-1.7), p=0.720 | -0.15 (-2.01-1.7), p=0.871 | -0.42 (-1.41-0.58), p=0.415 | 0.22 (0.05–1.02), p=0.054 | 1.58 (0.57–4.36), p=0.380 |
| South America | 1.18 (-0.48-2.84), p=0.165 | 0.9 (-0.44-2.24), p=0.187 | -0.06 (-1.4-1.29), p=0.932 | 0.79 (0.29–2.15), p=0.645 | 0.88 (0.35–2.18), p=0.776 |

**Supplementary Table 2: Referral metrics by continent.**

Generalized estimating equation (GEE) results comparing continents with Europe. For continuous outcomes (age at epilepsy onset, age at surgery, epilepsy duration), entries represent estimated differences in years with 95% Confidence Intervals (CIs); negative values indicate a lower mean than Europe; positive values a higher mean. For proportional outcomes (surgery before pharmacoresistance; surgery in the first year of life), entries are odds ratios (ORs) with 95% CIs; OR <1 indicates a lower rate and OR >1 a higher rate than Europe. The intercept represents Europe’s mean (continuous outcomes) or baseline odds (proportional outcomes). Statistically significant values (p < 0.05) are shown in bold. Africa was excluded due to small sample size (n = 17).

| Continent | Prior surgery | Prior resection | Prior disconnection | Prior neuromodulation |
| --- | --- | --- | --- | --- |
| (Intercept) | 0.21 (0.15–0.3), p<0.001 | 0.11 (0.08–0.16), p<0.001 | 0.02 (0.01–0.05), p<0.001 | 0.05 (0.02–0.12), p<0.001 |
| Asia | **0.2 (0.07–0.57), p=0.003** | not estimable | 0.29 (0.06–1.37), p=0.119 | **0.04 (0–0.42), p=0.007** |
| North America | **1.91 (1.07–3.41), p=0.029** | 1.41 (0.84–2.38), p=0.195 | 1.9 (0.64–5.62), p=0.245 | 2.69 (0.99–7.29), p=0.052 |
| Oceania | 1.29 (0.8–2.09), p=0.293 | 1.74 (0.78–3.85), p=0.175 | 2.18 (0.69–6.89), p=0.185 | not estimable |
| South America | 20.3 (0.12–3428.54), p=0.250 | 0.63 (0.33–1.23), p=0.177 | 2.04 (0.76–5.49), p=0.157 | 1.26 (0.38–4.22), p=0.706 |

## **Supplementary Table 3: Prior neurosurgery by continent.**

Generalized estimating equation (GEE) odds ratios (ORs) with 95% Confidence Intervals (CIs) for prior surgery, prior resection, prior disconnection, and prior neuromodulation, comparing each continent with Europe. The intercept represents Europe’s baseline odds. Statistically significant values (p < 0.05) are shown in bold. Africa was excluded due to the small sample size (n = 17).

| term | Estimate | 95% CI | p-value |
| --- | --- | --- | --- |
| (Intercept) | 4.50 | (3.94–5.06) | <0.001 |
| Reoperation rate | **5.50** | **(2.75–8.28)** | **<0.001** |

**Supplementary Table 4: Association Between Reoperation Rate and Epilepsy Duration.**

Generalized estimating equation (GEE) estimates describing the relationship between center-level reoperation rate and mean epilepsy duration (years). The positive coefficient indicates that higher reoperation rates are associated with longer epilepsy duration. Each 1% increase in reoperation rate corresponds to 0.05 years (~0.66 months) longer duration; a 10% increase corresponds to ~6.6 months longer duration (p < 0.001).

| Continent | History of IESS | History of DEE-SWAS | History of LGS |
| --- | --- | --- | --- |
| (Intercept) | 0.21 (0.17–0.26), p<0.001 | 0.06 (0.05–0.09), p<0.001 | 0.07 (0.04–0.12), p<0.001 |
| Asia | 0.54 (0.22–1.35), p=0.188 | 0.28 (0.05–1.66), p=0.162 | 0.56 (0.15–2.06), p=0.381 |
| North America | 0.61 (0.26–1.43), p=0.259 | **0.28 (0.13–0.61), p=0.001** | 1.72 (0.81–3.66), p=0.158 |
| Oceania | 0.95 (0.34–2.59), p=0.913 | not estimable | 0.73 (0.3–1.78), p=0.491 |
| South America | 0.83 (0.56–1.22), p=0.336 | **0.64 (0.46–0.88), p=0.007** | **3.89 (2.12–7.15), p<0.001** |

## **Supplementary Table 5: History of syndromes.**

Generalized estimating equation (GEE) odds ratios (ORs) with 95% Confidence Intervals (CIs) for documented history of infantile epileptic spasms syndrome (IESS), developmental and epileptic encephalopathy with spike-and-wave activation in sleep (DEE-SWAS), and Lennox–Gastaut syndrome (LGS), comparing each continent with Europe. OR <1 indicates a lower rate; OR >1 a higher rate than Europe. The intercept reflects Europe’s baseline odds. Statistically significant findings (p < 0.05) are shown in bold. Africa was excluded due to the small sample size (n = 17).

| Continent | IESS | DEE-SWAS | LGS |
| --- | --- | --- | --- |
| (Intercept) | 0.05 (0.03–0.07), p<0.001 | 0.02 (0.01–0.05), p<0.001 | 0.08 (0.05–0.12), p<0.001 |
| Asia | 1.43 (0.58–3.55), p=0.440 | 0.26 (0.07–1.01), p=0.052 | 0.73 (0.24–2.22), p=0.584 |
| North America | 1.09 (0.37–3.21), p=0.875 | 0.78 (0.28–2.19), p=0.637 | 1.71 (0.85–3.46), p=0.135 |
| Oceania | 0.27 (0.07–1.04), p=0.057 | 0.52 (0.07–4.14), p=0.540 | 0.7 (0.3–1.67), p=0.425 |
| South America | 0.77 (0.25–2.39), p=0.656 | 0.78 (0.2–3.06), p=0.724 | not estimable |

**Supplementary Table 6: Syndromes at the time of presurgical evaluation**.

Generalized estimating equation (GEE) odds ratios (ORs) with 95% Confidence Intervals (CIs) for infantile epileptic spasms syndrome (IESS), developmental and epileptic encephalopathy with spike-and-wave activation in sleep (DEE-SWAS), and Lennox–Gastaut syndrome (LGS) at presurgical evaluation, comparing each continent with Europe. OR <1 indicates a lower prevalence and OR >1 a higher prevalence than Europe. The intercept reflects Europe’s baseline odds. Statistically significant findings (p < 0.05) are shown in bold. Africa was excluded due to the small sample size (n = 17).

| Continent | All |
| --- | --- |
| (Intercept) | 4.08 (3.47-4.69), p<0.001 |
| Asia | **0.86 (0.03-1.68), p=0.042** |
| North America | 0.78 (-0.05-1.61), p=0.065 |
| Oceania | 0.72 (-0.44-1.87), p=0.222 |
| South America | -0.1 (-0.94-0.74), p=0.816 |

**Supplementary Table 7: Diagnostic modalities used per patient by continent.**

Generalized estimating equation (GEE) estimates comparing the number of diagnostic modalities used per patient across continents, with Europe as the reference. Entries represent estimated differences with 95% confidence intervals (CIs); negative values indicate fewer modalities and positive values more modalities than Europe. The intercept reflects Europe’s baseline estimate. Statistically significant results (p < 0.05) are shown in bold. Africa was excluded due to the small sample size (n = 17).

| Continent | fMRI  (language  or motor) | Interictal  FDG PET | Other  PET | Ictal  SPECT | MEG | Wada test | Genetic  testing | EEG-fMRI | MRI  postprocessing | Source  localization | hd-EEG |
| --- | --- | --- | --- | --- | --- | --- | --- | --- | --- | --- | --- |
| (Intercept) | 0.23 (0.16–0.34), p<0.001 | 0.41 (0.24–0.71), p=0.001 | 0.06 (0.01–0.32), p<0.001 | 0.06 (0.02–0.15), p<0.001 | 0.04 (0.01–0.1), p<0.001 | 0.02 (0.01–0.05), p<0.001 | 0.63 (0.37–1.09), p=0.100 | 0.01 (0–0.04), p<0.001 | 0.47 (0.19–1.13), p=0.090 | 0.03 (0.01–0.08), p<0.001 | 0.05 (0.02–0.13), p<0.001 |
| Asia | **0.11 (0.03–0.38), p<0.001** | **9.04 (3.97–20.62), p<0.001** | 1.48 (0.14–15.49), p=0.742 | 1.85 (0.4–8.43), p=0.429 | 2.75 (0.45–16.79), p=0.274 | not estimable | 2.35 (0.8–6.9), p=0.121 | 0.21 (0.02–2.74), p=0.235 | not estimable | not estimable | not estimable |
| North America | **2.9 (1.49–5.65), p=0.002** | **3.46 (1.51–7.91), p=0.003** | 0.08 (0.01–1.04), p=0.053 | **4.88 (1.62–14.71), p=0.005** | **17.13 (4.87–60.27), p<0.001** | 2.19 (0.53–9.01), p=0.277 | 1.81 (0.92–3.57), p=0.086 | not estimable | 0.55 (0.1–3.1), p=0.496 | 0.42 (0.06–3.12), p=0.399 | 0.16 (0.02–1.2), p=0.075 |
| Oceania | 1.18 (0.38–3.62), p=0.774 | **9.56 (2.4–38.1), p=0.001** | not estimable | 1.4 (0.56–3.5), p=0.469 | 0.34 (0.05–2.37), p=0.275 | not estimable | 0.73 (0.33–1.61), p=0.439 | not estimable | 1.88 (0.17–20.76), p=0.605 | 2.8 (0.54–14.63), p=0.222 | not estimable |
| South America | **0.07 (0.01–0.61), p=0.016** | **0.35 (0.14–0.91), p=0.031** | 1.05 (0.16–6.87), p=0.956 | not estimable | not estimable | 0.85 (0.2–3.59), p=0.825 | 1.27 (0.46–3.5), p=0.644 | not estimable | not estimable | not estimable | 0.9 (0.09–9.15), p=0.930 |

**Supplementary Table 8: Use of specific presurgical investigations by continent.**

Generalized estimating equation (GEE) odds ratios (ORs) with 95% Confidence Intervals (CIs) for individual modalities—functional MRI (language/motor), interictal fluorodeoxyglucose PET (FDG-PET), other PET, ictal single-photon emission computed tomography (SPECT), magnetoencephalography (MEG), Wada test, genetic testing, EEG–fMRI, MRI post-processing, EEG source localization, and high-density EEG—comparing each continent with Europe. OR <1 indicates lower use; OR >1 higher use than Europe. The intercept reflects Europe’s baseline odds. Statistically significant values (p < 0.05) are shown in bold. Africa was excluded from the analysis due to the small sample size (n = 17).
